# Computational Investigation of the Chemical Bond between An(III) Ions and Soft-Donor Ligands

**DOI:** 10.1021/acs.inorgchem.4c03924

**Published:** 2025-03-21

**Authors:** Sabyasachi Roy Chowdhury, Naomi Rehberg, Bess Vlaisavljevich

**Affiliations:** † Department of Chemistry, University of Iowa, Iowa City, Iowa 52242, United States; ‡ Department of Chemistry, University of South Dakota, Vermillion, South Dakota 57069, United States

## Abstract

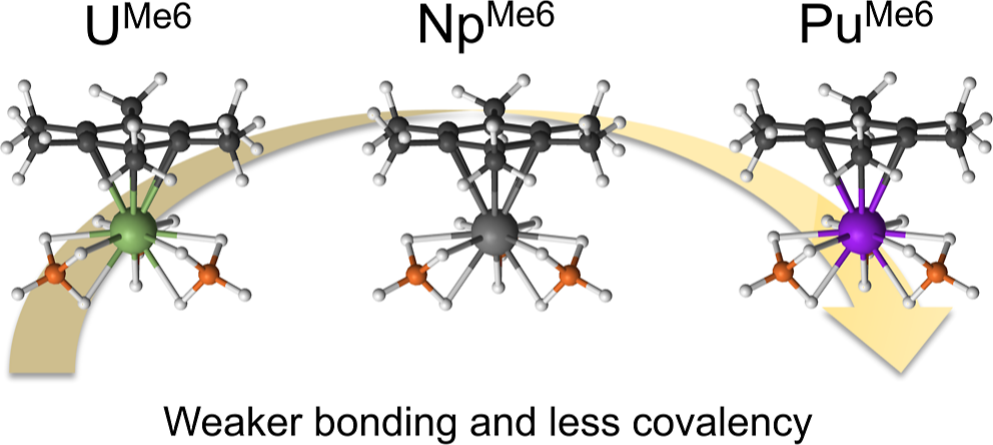

The chemical bonding of actinide ions with arene and borohydride ligands is explored via quantum chemical methods to understand how the transuranium elements interact with soft-donor ligands. Specifically, the  complexes (An = U, Np, and Pu) and their reduced congeners are studied. Density functional theory (DFT) shows that the metal–ligand interactions in the neutral complexes are governed by electrostatic interactions. Both DFT and complete active space (CASSCF) results show that as one moves from U to Pu, the 5f-orbitals are stabilized leading to a poorer energy match with the ligand orbitals. This contributes to progressively weaker metal-arene and metal-borohydride interactions across the series due to a decrease in energy-driven covalency. A reduction in orbital contributions to bonding is obtained for the transuranium-arene interactions as well. Upon reduction, the arene is reduced, forming a δ-bond. This causes the An–arene distances to contract by 0.1–0.2 Å compared to the neutral complexes. The ground state is assigned as the intermediate-spin state where the arene radical is antiferromagnetically coupled to the metal-centered f-electrons in Np and Pu. On the other hand, the ferromagnetically and antiferromagnetically coupled states are close in energy in the uranium complex, but do not mix when spin–orbit coupling is included using a state-interaction approach (SO-CASPT2). The population of the CASSCF δ*-antibonding natural orbital increases from U to Pu consistent with the increased An−arene distances, weaker interactions, and decreasing covalency across the series. Although the An–B distance increases by ca. 0.06 Å upon reduction, both the neutral and reduced species involve an An(III)–borohydride bond and as such are qualitatively similar. The Np complexes can be assigned to have slightly weaker bonding than the uranium analogs but are overall “uranium-like”. The Pu complexes are predicted to have less covalent contributions to bonding in both the Pu–arene and Pu–borohydride interactions; however, the Pu–arene interaction is predicted to be particularly weak.

## Abstract 2

The chemical bonding of actinide ions with arene and borohydride ligands is explored via quantum chemical methods to understand how the transuranium elements interact with soft-donor ligands.

## Introduction

The investigation of chemical bonding and the associated properties of actinide complexes is of interest to establish ligand design choices associated with separations relevant in the nuclear industry. A broad spectrum of work has shown the importance of orbital interactions in metal–ligand bonding, placing the majority of actinide coordination complexes at an intermediate position between highly covalent transition-metal complexes and highly ionic lanthanide complexes.^1−9^ Bursten defined the classic actinide−ligand bonding picture in his FEUDAL model which states that the f-orbitals are essentially unaffected by the ligand field, while the d-orbitals accommodate the ligands.^10^ However, actinide coordination complexes can exhibit non-FEUDAL chemical bonding where the 5f-orbitals form covalent bonds with the ligands,^11^ including cases where this effect is driven by the f-orbitals having the correct symmetry to mix with ligand orbitals. This can also involve bonding with contributions from both the 5f- and 6d-orbitals of the metal ions,^11−23^ and can lead to bonding motifs not accessible with d-block metals.^24−27^

By exploiting subtle bonding differences, actinide ions can be selectively coordinated over lanthanide ions when soft-donor atoms like nitrogen and sulfur are included in ligand design. The soft-donor ligand orbitals are more diffuse and polarizable compared to oxygen-based ligands. Likewise, the 5f-orbitals extend further than the lanthanide 4f-orbitals.^28−30^ Outside of separations science, researchers are also interested in stabilizing new metal–ligand interactions to tune the properties and oxidation states of the metal ions. Fortunately, soft-donor ligands are not limited to nitrogen and sulfur-donating complexes but include any highly polarizable ligand. Take for example halide ligands, which become more polarizable as one moves down the group. Chloride and bromide have reported polarizabilities of 3.0 and 4.158 Å^3^, respectively, and are considered soft-donor ligands.^31^ This work focuses on actinide interactions with arene and borohydride ligands. Arene ligands are extremely polarizable electron reservoirs (the polarizability of benzene is 9.959 Å^3^).^32^ Although less polarizable than an arene, the  borohydride ligand has a reported polarizability of 3.9 Å^3^, which falls between chloride and bromide.^31^ Both arenes and borohydrides have been used as soft-donor ligands to support interactions with uranium in the +3 oxidation state or lower.^30,33−40^

First consider the arene and borohydride ligands individually. Actinide-arene interactions are an example of ligands where non-FEUDAL bonding has been reported. The antibonding orbitals of the arene moiety can engage in π, δ, or ϕ-type overlap with both the f- and d-orbitals of the metal ions due to symmetry allowed interactions.^26,41−52^ The arene acts as an electron reservoir stabilizing metal ions in lower oxidation states.^38,51,53−56^ Moreover, lower oxidation states and lighter actinides have 5f-orbital energies that are closer to the unoccupied arene π-orbitals leading to stronger covalent interactions.^36,37^ Most of the actinide-arene complexes reported to date include uranium and can be categorized as tethered,^29,30,51,57,58^ sandwich,^38,53,59,60^ or inverse-sandwich complexes.^40,48,49,61^ However, stabilizing interactions between hard-acid f-block elements with soft-donor ligands like arenes can be challenging. Nevertheless, synthetic strategies have been successful by incorporating anionic ligands as an anchor to bind with the metal ion, supporting the metal-arene interaction.^21,29,38,51−53,57−60,62−65^ For example, Meyer and co-workers have reported tethered actinide-arene complexes using aryloxide ligands,^51,52,58,62^ while Fortier and co-workers have characterized complexes using anilide-based anchor ligands.^29,66^ Mazzanti and co-workers used siloxide ligands to stabilize lanthanide and actinide-arene complexes.^55,56,67,68^ Additionally, Arnold and co-workers designed a macrocyclic ligand to synthesize several uranium-arene sandwich complexes by employing pyrrolide groups as the anchor in their ligand.^59,60^ This approach was also used later to synthesize transuranium-arene complexes.^22,23^

On the other hand, ligands with anilide groups,^40^ kitimide groups,^40,48^ and ligands with uranium–carbon multiple bonds have been used to synthesize actinide-arene inverse-sandwich complexes.^61^ These complexes are stabilized by δ-bonding interactions in which the f-orbitals on both uranium centers have the correct symmetry to overlap with the arene π-system, another example of non-FEUDAL bonding.^40,48,61^ The oxidation state of the metal and total charge on the arene depend on the nature of this interaction and have been the focus of a handful of computational studies.^49,61,69^ Despite extensive structural exploration of actinide-arene complexes as a whole, little work has focused on the reported terminal arene complexes involving ancillary tetrachloroaluminate (AlCl_4_^–^) or borohydride (BH_4_^–^) ligands.^35,70−74^

Of the reported complexes,  drew our attention since it not only includes the highly polarizable hexamethylbenzene ligand, but also soft-donor borohydride ligands.^35^ Interactions between actinides centers and borohydride ligands have been used in the nuclear industry for their high volatilities. They are of fundamental interest as researchers seek to control hydrogen positioning around the metal.^75−78^ Specifically, the first actinide borohydride, tetravalent , was reported during the Manhattan Project.^79^ Due to its high volatility, it was used in separating enriched U-235 from abundant U-238 to minimize the production of radiochemical waste during the separation process.^75−78^ Following the synthesis of , the transuranium analogs  and  were reported.^80^ Among the transuranium borohydrides,  stands out as a unique example of a molecular plutonium hydride. Understanding discrete Pu–H interactions can provide insight into solid-state plutonium hydride phases, which are not well understood despite being problematic corrosion products that form as plutonium metal is exposed to hydrogen gas.^81^ Specifically, the presence of hydride-coated plutonium surfaces poses challenges for both long-term storage and nuclear waste management.^82,83^ Structural characterization of plutonium hydride surfaces is challenging; therefore, synthesizing molecular models allows one to characterize well-defined Pu–H interactions. Recently, the first solid-state characterization of a Pu(III) borohydride was reported,^34^ highlighting the scarcity of compounds with actinide-hydride bonds in lower oxidation states.

**Figure 1:**
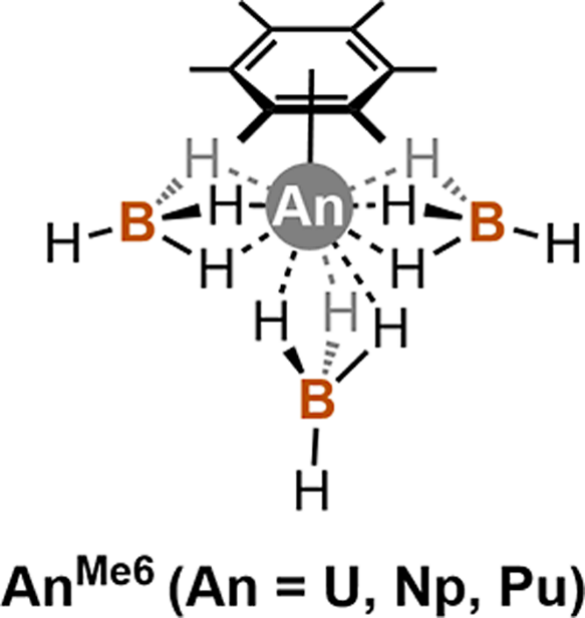
Molecules studied in this work. The uranium complex has been characterized in the solid state and by theory,^33,35^ while the Np and Pu complexes have not been characterized.

The growing interest in synthetically modeling such interactions in transuranium coordination chemistry has led to the synthesis of new transuranium-arene and transuranium-borohydride complexes involving both Np and Pu;^22,23,34,66^ however, such synthetic efforts remain challenging, expensive, and time-consuming. Detailed theoretical investigations into actinide–ligand bonding can provide insights both to guide future synthetic models and also to systematically explore the bonds in their own right. However, previous research on actinide-arene interactions has focused on bis-benzene actinide complexes and matrix isolation experiments involving actinide atoms and aromatic molecules.^84−87^ Some combined experimental and theoretical studies have examined U–arene bonding,^51^ including the influence of hapticity on the nature of the interaction,^36^ but the role of substituents on the terminal arene complexes was only recently investigated by us.^33^ We studied the nature of the bonding between trivalent uranium and terminal arenes in a series of  complexes (including the U complex with hexamethylbenzene in Figure 1). The presence of electron-donating groups on the arene increased the strength of the interaction with uranium, since it is a primarily electrostatic bond. Furthermore, we showed that upon reduction arene distortion is indicative of an arene radical; however, the lack of distortion is not sufficient to assign a metal-centered reduction. Regardless of spin state and structural hallmarks, the reduced species was best described as an U(III)−arene radical.^33^

This work extends the study to transuranium chemistry (Figure 1) by exploring the  series (denoted **An^Me_6_^**) where An = U, Np, and Pu.^88^ We seek to determine if the nature of bonding changes as a function of metal choice, both for the arene and the borohydride, to enhance our understanding of how uranium and transuranium elements interact with soft-donor ligands.

## Computational Details

Geometry optimizations of the complexes were carried out in the gas phase using the PBE0 functional,^89^ taking the crystal structure of  (labeled  herein) as the initial geometry.^35^ The choice of functional was made for consistency with prior work; a choice supported by comparisons with a variety of functionals.^33^ The actinide ions were treated with the def-TZVP basis set and scalar relativistic effects are recovered with the corresponding effective core potential (ECP).^90,91^ The def2-TZVP basis set was employed for the rest of the elements.^92^ For each geometry optimization, the convergence threshold of the Cartesian gradient was set to 1 × 10^–4^ a.u. All geometries were confirmed as minima by means of harmonic vibrational analysis. The resolution of identity (RI) approximation was also employed for integral evaluation and grid size was selected with the multiple grid (m4) option.^93^ The impact of an implicit solvent model was tested (Tables S1–S3), but found not to impact the geometries or the nature of the DFT orbitals. These DFT calculations were performed with the Turbomole program package V7.3.^94^

To characterize metal–ligand bonding in the previously described DFT computations, topological analysis of the electron density was performed with Bader’s Quantum Theory of Atoms in Molecules (QTAIM)^95^ as implemented in the Multiwfn 3.8 program.^96^ The bonding was further characterized by performing energy decomposition analysis (EDA)^97^ as implemented in the Amsterdam Density Functional (ADF) program.^98^ The PBE functional,^99,100^ together with the Grimme’s D3 dispersion correction,^101^ was selected. The TZP basis set was used on all atoms with no frozen core electrons. Relativistic corrections were taken into account by employing the scalar relativistic zero-order regular approximation (ZORA).^102^

DFT optimized geometries were subjected to multiconfigurational complete active space (CASSCF)^103^ calculations followed by second-order perturbation theory (CASPT2).^104,105^ In the CASSCF calculations, an active space of n electrons in 13 orbitals, denoted (n*e*, 13*o*), was used for the neutral complexes where the seven 5f-orbitals and six arene π-orbitals are included, along with the corresponding n electrons (Figures S1–S3). For the reduced complexes, the active space was extended to include both the additional electron and the five 6d-orbitals, resulting in an active space of (n + 1*e*, 18*o*) (Figures S4–S9). In higher oxidation states such as An(III), it is generally less important to include the 6d-orbitals in the active space compared to computations for systems in lower oxidation states. This assumption was previously tested for uranium,^33^ supporting that the larger active space is important in the reduced complex. For , the ground state is 3-fold degenerate; therefore, the CASSCF calculation was state-averaged over three sextet states. In the other complexes, only the lowest energy state of the respective spin-multiplet was computed.

In the CASPT2 calculations, the zeroth-order Hamiltonian included both an IPEA shift of 0.25 au^106^ and an imaginary shift of 0.2 au. For , the extended-multistate CASPT2 (XMS-CASPT2) method was used (3 states included in state averaging).^107^ All other CASPT2 calculations are state specific. Scalar relativistic effects were included at the CASSCF/CASPT2 level using the second-order Douglas-Kroll-Hess (DKH) Hamiltonian^108^ and relativistic all-electron ANO-RCC basis sets.^109^ Specifically, we use a contraction of [9s,8p,6d,4f,2g,1h] for the actinides, [4s,3p,2d,1f] for boron and carbon atoms in the first coordination sphere, [3s,2p,1d] for the peripheral carbon atoms, and [1s] for hydrogen atoms. Cholesky decomposition in conjunction with local-exchange screening was used to reduce the computational cost of integral evaluation.^110^ The energy was converged to 10^–8^ a.u. The impact spin–orbit (SO) coupling was assessed via the SO-CASPT2 approach using a state-interaction Hamiltonian (see the Spin–Orbit Coupling Section in the Supporting Information for details).^111^ All multireference calculations were carried out using the OpenMolcas software suite V24.02.^112^

## Results and Discussion

Prior to the discussion of the transuranium complexes, a summary of the previously reported U(III)−arene complex,  (Figure 1), is given.^33^ The uranium–arene bond was characterized as a weak bond, predominantly electrostatic in nature. However, the bond can be strengthened by selecting electron-rich ligands such as hexamethylbenzene. Weaker interactions were obtained for arene ligands with electron-poor π-systems. Reducing the complex to yield a putative U(II) complex exhibited a characteristic δ-bond. Arene distortion was shown to be an unreliable metric for assigning oxidation state. Specifically, arene radical character was present even when the arene geometry remained planar upon reduction. Here, we explore how both the metal-arene and metal-borohydride interactions change across the early actinides.

### Molecular Structure and Bonding in Neutral  Species

Optimized geometries for  were in good agreement with experiment;^33^ however, no experimental comparison is possible for the transuranium elements. The average PBE0 An–arene bond distance increases from U(III) to Pu(III) by 0.07 Å (Table 1). Specifically, the average U–arene distance in  was 2.903 Å, which increased to 2.928 and 2.976 Å for  and , respectively. One may be surprised by this result since the ionic radius of the elements decreases across the series: U(III) is 1.025, Np(III) is 1.01, and Pu(III) is 1.^113^ Shorter bond distances for smaller ionic radii have been reported for a variety of U, Np, and Pu complexes in different ligand environments and oxidation states.^18,114−120^ On the other hand, the calculations herein are consistent with structures reported for the U, Np, and Pu tethered-arene complexes synthesized by Murillo et al. where the average U–arene distance was shorter at 2.90 Å compared to the longer distances of 2.91 and 2.93 Å for the Np and Pu arene complexes, respectively.^66^ The elongation observed for these tethered transuranium complexes is smaller but the overall trend is consistent with our values and both systems have predominantly ionic bonds.

**Table 1:** Selected DFT (PBE0) Bond Distances in Å^a^

complex	An–arene	An–B	An–H
(33)	2.903 (2.932)	2.525 (2.568)	2.337
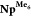	2.928	2.510	2.323
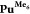	2.976	2.513	2.329

a Experimental bond distances are in parentheses.^35^

Comparing the molecular geometries for the borohydride ligands, we first note that all three  ligands coordinate in the expected κ^3^-coordination mode. The average U–B distance is 2.525 Å, which is 0.043 Å shorter than experiment. Note that the experimental U–B distances are not equivalent (2.482, 2.687, and 2.537 Å) with a mean of 2.568 ± 0.086 Å, whereas DFT yields structures with three equivalent U–B distances.^33^ Similarly, the calculated An–B distances in  and  are equivalent, with computed distances of 2.510 and 2.513 Å, respectively (Table 1). Although the Np–B and Pu–B bond distances are shorter than the U–B distances, the differences are too small (ca. 0.003 Å) to assign a trend. On the other hand, the calculated An–H distances show slightly more variation with metal choice and follow the expected trend based on actinide ionic radius. The average An–H bond distances are 2.337, 2.323, and 2.329 Å for , , and , respectively (Table 1).

To further explore the An–arene and An–borohydride interactions, topological analysis using Bader’s Quantum Theory of Atoms in Molecules (QTAIM) was performed.^95^ Bond critical points (BCPs) between An–C_arene_ and An–B atoms in the complexes exhibit a positive Laplacian of the electron density, ∇^2^ρ, and a negative total electronic energy density, E(r), consistent with dative interactions (Tables S4 and S5). However, differences in covalent contributions even in highly polarized bonds can lead to small but meaningful changes in bonding energies. A higher electron density, ρ, at the BCP could indicate overlap-driven covalency, whereas a higher delocalization index, δ, could arise for cases where energy degeneracy-driven covalency dominates. Moving from  to , both  and  decrease (Table 2), consistent with the increase in An–arene distances observed across the series (Table 1). Although the U(III)–arene bond is dominated by electrostatic interactions, the covalent contributions decrease from U to Pu due to the longer bond distance and, in turn, lower . Additionally, the stabilization of the metal 5f-orbitals leads to a less favorable energy overlap with the arene π-system (Table S3 and Figure S10). Based on these values, both orbital-driven and degeneracy-driven covalent interactions decrease in the actinide-arene interactions from U to Pu.

**Table 2:** Average Electron Density ρ, at the Bond Critical Points and the Average Delocalization Index δ, Along the An(III)–Arene and the An(III)–B Bonds^a^

complex	ρ_An–C_arene__	δ_An–C_arene__	ρ_An–B_	δ_An–B_
(33)	0.030	0.157	0.054	0.158
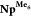	0.027	0.131	0.054	0.154
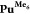	0.024	0.107	0.052	0.144

a All values are expressed in atomic units.

On the other hand, the interaction between the actinide ion and borohydride ligand yields values of similar magnitudes (ca. 0.05 au) for the electron density, ρ_An–B_, across the series. This suggest that there is no change in the orbital-driven covalent interactions across the series for borohydride ligands. Changes in δ_An–B_ are obtained arising from the stabilization of the 5f-orbitals across the series (Table 2). An analogous observation was recently reported by one of the authors for U(III) and Pu(III) borohydride complexes where ρ was a similar magnitude for both actinides but the δ value was larger in U than Pu.^34^ In the cited study, only U and Pu are characterized, but Np is included herein and the δ value is slightly smaller than U, supporting its assignment as more “uranium-like” than “plutonium-like”. Although no bond critical point was detected between the metal ions and hydrogen atoms, non-negligible values of δ_An–H_ are noted for all three complexes and the average δ value gradually decreases from  to . These values suggest that degeneracy-driven covalent interactions also decrease in the actinide-hydride interactions from U to Pu (Tables S6–S8).

While QTAIM analysis supports the assignment of primarily electrostatic actinide−ligand interactions with decreasing covalent contributions moving from U to Pu, these interactions can also be examined via energy decomposition analysis (EDA). To perform EDA, the molecule is first partitioned into two fragments. The total interaction energy between these fragments, Δ*E*_Int_, includes contributions from Pauli repulsion (Δ*E*_Pauli_) and the attractive orbitalic (Δ*E*_Orb_), electrostatic (ΔV_Elect_), and dispersion (Δ*E*_Disp_) interactions (eq 1).^97^
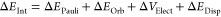
1

**Figure 2:**
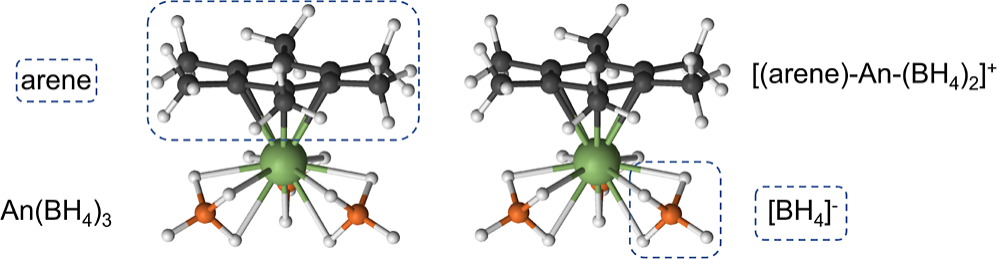
Molecular fragments considered in the EDA calculations. The fragment for the ligand of interest (arene on the left, borohydride on the right) is shown in the dashed rectangular box, and the remainder of the molecule is taken as the other fragment.

Since the total interaction energy varies, the attractive portion is reported as a percent contribution for each type. To study the An–arene and An–BH_4_ interactions separately, two different EDA calculations were performed for each complex where the fragments were (1)  interacting with the arene and (2)  interacting with one  ligand (Figure 2).

When considering the arene as a fragment (Table 3), the total interaction energy for  was −48.7 kcal/mol with 47.9% orbitalic, 44.8% electrostatic, and 7.2% dispersion contributions.^33^ On first glance, one may think the orbitalic, and in turn covalent, contributions to the interaction are the most important in contrast with the QTAIM results; however, this is not the case. Subsequent analysis with the extended transition state-natural orbital for chemical valence (ETS-NOCV) method showed that this large percentage is only reflective of the reorganization of metal-centered f-orbitals and not a result of charge reorganization between the ligand and metal.^33^ Therefore, this work focuses on how the total interaction energy and the electrostatic contributions vary across the series. Comparing Np to U, the total strength of the metal–ligand interaction becomes weaker by 3.4 kcal/mol (Table 3). While the percent electrostatic contribution remained nearly constant, a 1.1% decline in the orbitalic contribution was observed accompanied by a corresponding increase in the dispersion interaction of similar extent. Moving further along the series from Np to Pu, the total interaction energy was further reduced by 4.1 kcal/mol. A marginal increase in the electrostatic contribution (0.6%) was observed alongside a 2.1% increase in the dispersion contribution. However, the orbital contribution decreased by 2.7%.

**Table 3:** Energy Decomposition Analysis of the  Complexes Considering the Arene and  as the Two Fragments^a^

complex	Δ*E*_Int_	Δ*E*_Pauli_	Δ*E*_Orb_	Δ*V*_Elect_	Δ*E*_Disp_	Δ*V*_Elect_ + Δ*E*_Disp_
(33)	–48.7	77.2	–60.4	–56.4	–9.1	–65.5
			47.9%	44.8%	7.2%	52%
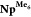	–45.3	63.9	–51.0	–49.1	–9.1	–58.2
			46.8%	44.9%	8.3%	53.2%
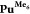	–41.2	55.5	–42.6	–44.0	–10.1	–54.1
			44.1%	45.5%	10.4%	55.9%

a All energies are in kcal/mol.

**Figure 3:**
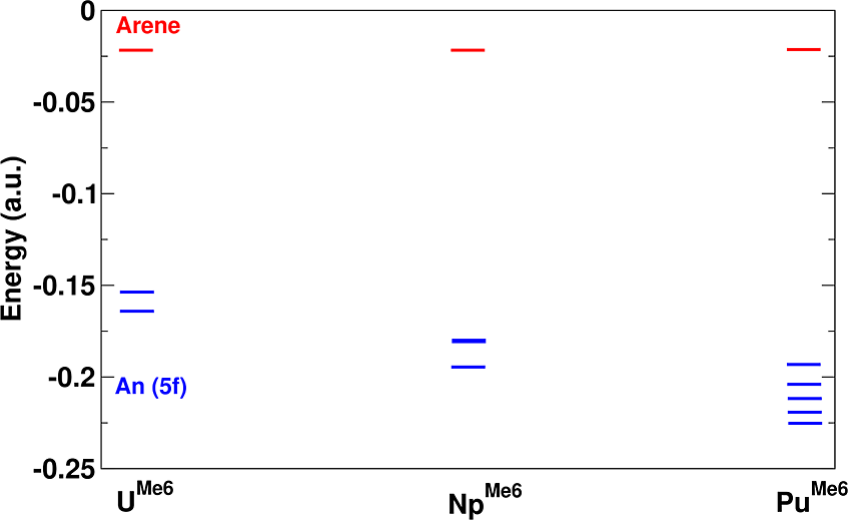
Comparison of the DFT Kohn–Sham orbital energies (α spin) of the complexes, obtained from EDA calculations. Arene orbitals are shown in red and An 5f-orbitals in blue.

In other words, progressing from U to Pu decreased the strength of the interaction between the actinide fragment and the hexamethylbenzene arene. This change was accompanied by a small increase in nonorbital contributions (Δ*V*_Elect_ + Δ*E*_Disp_) and a decrease in orbital contributions. Consistent with the QTAIM results, the bonding for all three systems is predominantly electrostatic with covalent contributions decreasing across the An series. Our earlier work explored the impact of modifying the substituents on the arene. As the number of methyl groups on the arene was increased, the U–arene Δ*E*_Int_ became more negative, indicating a stronger U–arene interaction.^33^ Comparing Δ*E*_Int_ across different U–arene complexes with  and , the strength of Δ*E*_Int_ in  is comparable to  and  whereas the Δ*E*_Int_ of  is similar in magnitude to the weak interaction observed for . Moreover, the Kohn–Sham orbital energies show the stabilization of the 5f-orbitals across the early actinides that lead to poorer energy matching with the arene π-system (Figure 3). Like QTAIM, EDA analysis shows metal-arene interactions for U and Np, while Pu has a weaker interaction.

Likewise, the interaction between the borohydride ligand and actinide fragment was examined using EDA. As expected, a larger total interaction energy was obtained given that charged fragments were used (Table 4). Nevertheless, the trend in how the interaction energy varies across the early actinides was consistent with the results obtained for the arene fragment. The total interaction energy decreases across the series with a value of −160.1 kcal/mol for the  complex (Table 4). The interaction is weaker by 2.3 kcal/mol for Np. When Np is replaced with Pu, the interaction energy was further destabilized by 4.3 kcal/mol. These interaction energies are dominated by electrostatic contributions which increase across the series from 67.8% to 68.7% to 69.3% for U, Np, and Pu, respectively. Only minor variations in the percentages of orbital and dispersion contributions are obtained. As with the arene ligand, the fragments with the heavier actinides had weaker interactions with the BH_4_^–^ ligand, despite an increase in the percent electrostatic interaction.

**Table 4:** Energy Decomposition Analysis of the  Complexes Considering  and  as the Two Fragments^a^

complex	Δ*E*_Int_	Δ*E*_Pauli_	Δ*E*_Orb_	Δ*V*_Elect_	Δ*E*_Disp_	Δ*V*_Elect_ + Δ*E*_Disp_
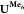	–160.1	105.7	–81.9	–180.2	–3.7	–183.9
			30.8%	67.8%	1.4%	69.2%
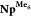	–157.8	105.2	–78.6	–180.6	–3.7	184.3
			29.9%	68.7%	1.4%	70.1%
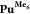	–153.5	101.2	–74.4	–176.5	–3.9	180.4
			29.2%	69.3%	1.5%	70.8%

a All energies are in kcal/mol.

**Figure 4:**
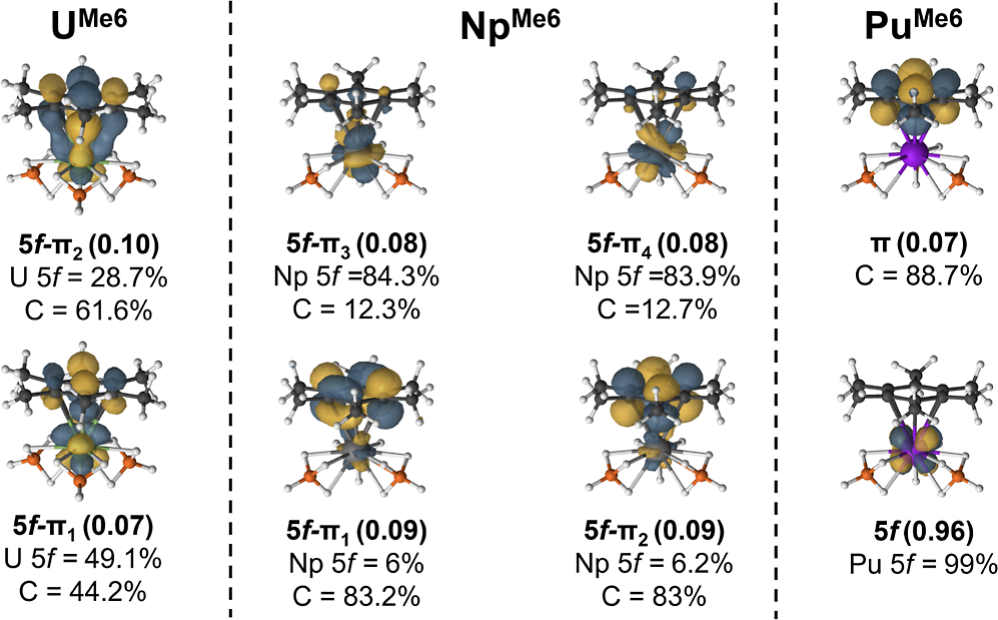
Comparison of the selected CASSCF natural orbitals in the neutral  complexes that show promise of forming a δ-bond upon reduction.

**Figure 5:**
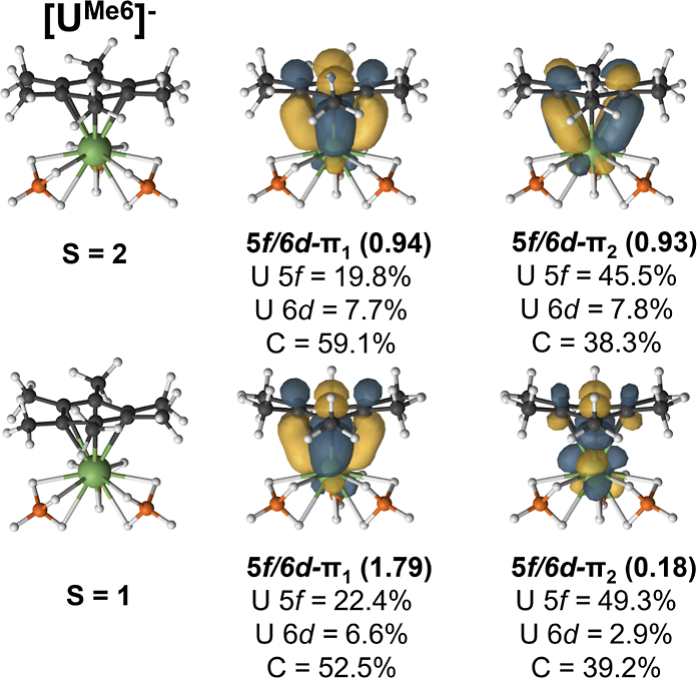
PBE0 molecular geometries and selected CASSCF natural orbitals of the  complexes. CASSCF occupation numbers are given below each natural orbital along with the percent contribution of metal and arene carbon atomic orbitals.

The three neutral complexes (, , and ) are best described by DFT as an An(III)-ion electrostatically coordinated to the hexamethylbenzene arene and borohydride ligands. All three neutral complexes were subsequently studied with CASSCF. Consistent with DFT, no δ-bond is observed in the neutral complexes. However, the lowest unoccupied CASSCF natural orbital in  exhibits mixing between the arene π-system and a uranium 5f-orbital (Figure 4, top left). Upon reduction, this orbital is occupied and forms a true δ-bonding interaction (Figure 5). Reduced mixing is observed in the analogous unoccupied orbital for the neutral Np complex and a more delocalized “δ-like” interaction is obtained (Figure 4). On the other hand, Pu shows a marked reduction in orbital mixing. Our goal was to determine whether a δ-bonding interaction would be obtained in the either of the reduced transuranium complexes and what impact, if any, this has on the nature of the ground state.

### Molecular Structure and Ground State Assignment in the Reduced Complexes

Before characterizing the bonding in the reduced complexes with CASSCF, the molecular geometries were optimized using DFT for all possible spin states. In the reduced  species, three spin states are accessible (*S* = 2, *S* = 1, and *S* = 0). Three spin states are also accessible in  (*S* = 5/2, *S* = 3/2, and *S* = 1/2). The reduction of  to  leads to four possible spin-states (*S* = 3, *S* = 2, *S* = 1, and *S* = 0) (Table 5). DFT predicts the high-spin quintet state as the ground state for ; however, a low-lying triplet state is only 1.5 kcal/mol higher. Likewise, two spin states lie close in energy for . The quartet state is the lowest in energy while the high-spin sextet state is 2.5 kcal/mol higher. The low-spin (doublet) state is well separated lying 23.3 kcal/mol higher. For , DFT predicts that the high-spin septet is the ground state with a quintet state 6.1 kcal/mol higher. The lower-spin triplet and singlet spin states are well separated. For all of the actinides and all of the spin states, the arene is reduced and the An ion remains in the +3 oxidation state (see Oxidation State Assignment Section in the Supporting Information). The two lowest energy spin states for the  complexes are (1) the arene radical ferromagnetically coupled to the f-electrons in the high-spin configuration or (2) the arene radical antiferromagenetically coupled to the f-electrons in an intermediate-spin configuration. Note that some spin contamination is obtained for intermediate-spin states (Table 5); however, the energy splitting between the low-lying spin states has also been computed with CASPT2. With the exception of  where spin–orbit coupling mixes the two lowest states (vide infra), DFT and CASPT2 predict the same ground state.

**Table 5:** Relative Energies of the Different Spin States of the Reduced Complexes^a^

complex	spin	Δ*E*^DFT^	Δ*E*^CASPT2^	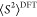	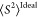
(33)	2	0.0	0.0	6.0	6
	1	1.5	2.5	2.4	2
	0	33.5	17.1	0.0	0
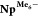	5/2	2.5	10.4	8.8	8.75
	3/2	0.0	0.0	4.5	3.75
	1/2	23.3	30.7	2.3	0.75
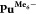	3	0.0	8.5	12.0	12
	2	6.1	0.0	6.9	6
	1	36.7	34.7	3.6	2
	0	79.2	53.5	0.0	0

a CASPT2 calculations are performed on the DFT optimized geometry for the spin state of interest. All energies are in kcal/mol.

Returning to the DFT structures, reduction induces a contraction in the An–arene distances. Larger contractions are obtained for the intermediate-spin states. For example, in the high-spin *S* = 5/2 state, the average Np–arene distance contracts by 0.11 Å, while a contraction of 0.17 Å is obtained for the *S* = 3/2 state (Table 6). Similar contractions are obtained for U and Pu. This is comparable to geometry changes reported for other tethered U–arene complexes in low oxidation states.^38,51,53−56^ For example, in the formally divalent [U{mes(OAr^Ad,Me^)_3_}]^−^ complex, the U–C range is 2.597–2.633 Å, while in the trivalent precursor it is 2.729–2.774 Å, a contraction of roughly 0.10–0.15 Å.^51^ Similarly, in trivalent [U(NHAr^iPr_6_^)_2_]^+^, the U–C range is 2.828–3.059 Å, while in the neutral divalent species it is 2.723–2.902 Å, a contraction of 0.10 Å upon reduction.^38^ To the best of our knowledge, no formally divalent uranium-terminal arene complexes have been reported, and both terminal or tethered Np and Pu arene complexes are exceedingly rare.^66^

**Table 6:** PBE0 Computed Selected Geometric Parameters of the  Complexes in Spin States for Which a Bonding Discussion is Included (Geometric Parameters for all Calculated Spin States are in Table S9)^a^

complex	spin	An–arene	An–B	book angle
(33)	2	2.709 (−0.194)	2.593 (0.068)	3.0
	1	2.703 (−0.200)	2.587 (0.062)	22.3
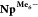	5/2	2.818 (−0.110)	2.600 (0.090)	11.0
	3/2	2.759 (−0.169)	2.572 (0.062)	15.1
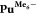	3	2.870 (−0.106)	2.634 (0.121)	3.0
	2	2.781 (−0.195)	2.580 (0.067)	15.4

a Deviation of bond distances from the neutral geometries are provided in the parentheses. Distances in Å. Angles in degrees.

In addition to bond distances, the arene exhibits subtle out-of-plane distortions (<1°) across the ipso-substituents of the aromatic ring for the neutral complexes (Table 6). Reduction of the system, not only changes the An–arene distances but can also induce a characteristic deformation of the arene. For instance, in the high-spin state of , an “open-book” arene conformation is observed with an out-of-plane angle of 11.0°, which increases to 15.1° in the intermediate-spin geometry. Alternatively, the arene appears nearly planar in  for the high-spin state (with only 3.0° deviation), while in the geometry for the intermediate-spin state, the arene exhibits a boat-like structure with an out-of-plane angle of 15.4°. This is consistent with previously reported structural changes in . Specifically, the optimized geometry for the high-spin state had a planar arene (with a 3.0° deviation), while the geometry for the intermediate-spin adapted an “open-book” arene conformation with an out-of-plane angle of 22.3°.^33^ Note that examples of tethered and sandwich uranium-arene complexes with both the planar^51,54^ and distorted^38,53,55,56^ arenes have been characterized upon reduction.

Single-point CASPT2 calculations were performed on the DFT geometries to further confirm ground state assignment (Tables 5, and S10–S12). In , DFT and CASPT2 ground state assignments are consistent with a quintet ground state (*S* = 2) and a low-lying triplet state (*S* = 1) (Table 5). On the other hand, the ordering of the states inverts for the triplet geometry where CASPT2 energies predict a triplet ground state with a low-lying quintet state. Although the DFT and CASPT2 spin-free energies are consistent for uranium, spin–orbit (SO)-CASPT2 states were computed by coupling only the lower energy spin-free states (Table S10). We emphasize that this is an approximate, yet computationally demanding, approach to include spin–orbit coupling for systems where large active spaces are required (see the Spin–Orbit Coupling Section in the Supporting Information). For , the lowest SO-CASPT2 states do not include significant mixing between the *S* = 2 and *S* = 1 spin-free states, confirming the state ordering in the spin-free manifold (Tables 5, and S10).

However, the CASPT2 spin-free states tell a different story with  (Table 5). CASPT2 energies computed on the DFT optimized geometries show that the intermediate-spin state (*S* = 3/2) is more stable than the high-spin state (*S* = 5/2) with a difference of 10.4 kcal/mol. With DFT, the same ground state spin is predicted but the energy difference was only 2.5 kcal/mol. Given the larger energy gap, the vertical excitation energies were computed on the DFT optimized structures for the two spin states. Unlike uranium, CASPT2 predicted that the intermediate-spin is lower in energy on both structures (Table S11). This was further supported by SO-CASPT2 (Table S11). Therefore, the ground state of  is assigned as the intermediate-spin state (*S* = 3/2).

Finally, CASPT2 energies were computed for the  complex. Recall that the DFT ground state is the high-spin ground state (*S* = 3) with the intermediate-spin state (*S* = 2) lying 6.1 kcal/mol higher in energy. Single-point CASPT2 calculations on the respective DFT geometries show the opposite: the intermediate-spin state is 8.5 kcal/mol more stable than the high-spin state (Table 5). On the high-spin geometry, CASPT2 vertical excitation energies show that the high-spin state remains the lowest in energy; however, the intermediate-spin state is nearly degenerate lying only 0.5 kcal/mol (or 167.7 cm^–1^) higher (Table S12). The SO-CASPT2 ground state on this structure consists of 37% septet and 61% quintet spin-free states (Table S12). On the intermediate-spin geometry, less mixing upon including spin–orbit coupling is observed with the intermediate spin-free state (*S* = 2) comprises 88% of the lowest energy spin–orbit state. Comparing the SO-CASPT2 energies on both structures, the geometry optimized for the intermediate-spin is lower by 7.5 kcal/mol (Table S13). This is in good agreement with the ground state assignment from the CASPT2 spin-free states where the energy difference was 8.5 kcal/mol. Therefore, we assign the ground state as the intermediate-spin state where, like in U and Np, spin–orbit effects should not impact subsequent bonding analysis.

### Electronic Structure and Bonding in the Reduced Complexes

The active electrons in the  complexes occupy the CASSCF natural orbitals as follows: six occupy the three π-orbitals of the arene, two to four (depending on An ion) occupy 5f-orbitals localized on the metal ion, and the remaining two electrons are distributed in δ-bonding and antibonding orbitals (Figures S4–S9). The differences in how the δ-orbitals are occupied impacts the strength of the metal-arene interaction. An effective bond order (EBO) can be computed from the natural orbital occupation numbers (NOONs) to quantify covalent contributions to bonding (eq 2).
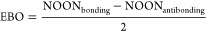
2

Recall that in , the adiabatic energy difference between the high-spin and intermediate-spin states was only 1.5 kcal/mol from DFT and 2.5 kcal/mol from CASPT2; therefore, bonding is discussed for both states. In the high-spin state, each of the δ-bonding orbitals is occupied by one electron (Figure 5). This pair of two one-electron bonds yields an EBO of 0.835. On the other hand, in the intermediate-spin state, one δ-bonding orbital is nearly doubly occupied with a NOON of 1.79 leading to an EBO of 0.805 (Table 7). Therefore, regardless of whether the arene radical is ferromagnetically or antiferromagnetically coupled to the f^2^ center, a δ-bond is present.

**Figure 6:**
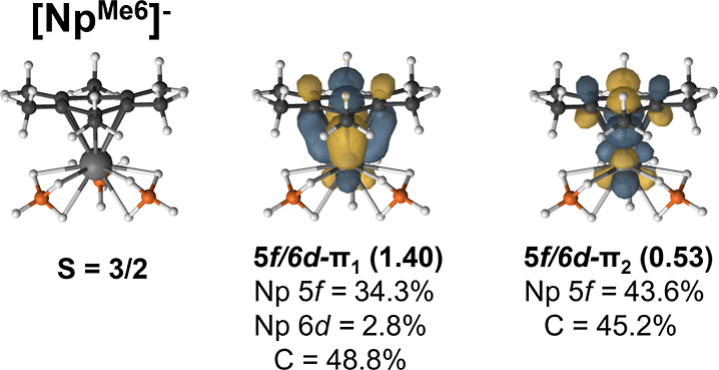
PBE0 molecular geometries and selected CASSCF natural orbitals of the  complexes. CASSCF occupation numbers are given below each natural orbital along with the percent contribution of metal and arene carbon atomic orbitals.

The ground state of  was assigned as the intermediate-spin state (*S* = 3/2). Two electrons are shared between a pair of δ-bonding and antibonding orbitals with NOONs of 1.40 and 0.53, respectively (Figure 6). The significant occupation of the δ*-orbital results in a weakening of the bond compared to uranium. The EBO is reduced to 0.435 which is approximately a bond order of one-half (Table 7). In , the intermediate-spin state (*S* = 2) was assigned as the ground state based on SO-CASPT2 computations. As was the case for Np, two electrons are shared between the δ-bonding and antibonding orbitals. In Pu, the antibonding orbital has an even higher NOON of 0.66 which further reduces the EBO to 0.305 (Table 7).

**Figure 7:**
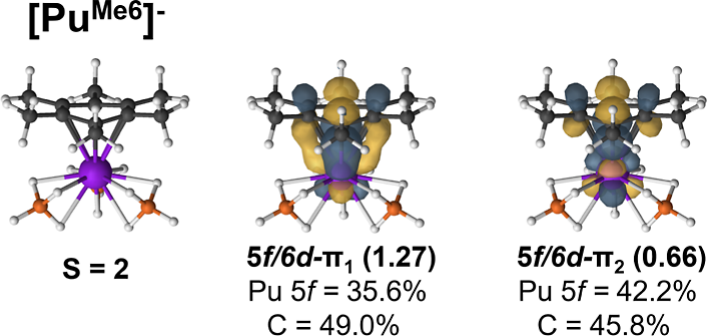
PBE0 molecular geometries and selected CASSCF natural orbitals of the  complexes. CASSCF occupation numbers are given below each natural orbital along with the percent contribution of metal and arene carbon atomic orbitals.

The differences in the nature of the δ-bonds across the series can also be described using the atomic orbital contributions to the natural orbitals. In the high-spin and intermediate-spin states of , both the 5f- and 6d-orbitals contribute to bonding (Figure 5). The total uranium contribution from the 5f-orbitals is ca. 20% while the contribution from the 6d-orbitals is ca. 8%. The 5f-orbitals decrease in energy across the series; however, their contribution to the lower-energy δ-bonding orbital is higher for the transuranium elements increasing to 34.3% and 35.6% in  and , respectively (Figures 6 and 7).

Simultaneously, the 6d-participation decreases from U to Np, and no 6d-participation is present in the  complex. These bonding differences are consistent with the molecular geometries and the FEUDAL model that suggests that it is the changes in 6d-contributions to bonding that are the driving force, despite the fact that the 5f-orbitals are allowed to mix by symmetry. In the intermediate-spin , the average An–arene distance was 2.703 Å, which increased to 2.759 Å for  and 2.781 Å in . This can be explained by the observed drop in the EBO from 0.805 to 0.305 across the series and the reduction in 6d-participation (Table 7). Consequently, the An–arene bond weakens from an effectively single δ-bond to the weak interaction in the Pu complex. This is also reflected in the CASPT2 computed electron affinity values (Table S14), which gradually decrease from uranium to plutonium.

Since reduction led to the formation of an arene radical, this section has focused on the actinide-arene bonding; however, the reduction of the complex could also impact the An(III)−borohydride interaction. The An–B distances elongate upon reduction by 0.06 Å in the ground state structures (Table 6), but the differences among the spin states are subtle compared to the more dramatic contractions observed in the An–arene distances. Moreover, the An–B interactions are much more electrostatic compared to the An–arene interactions (Table 4). A comparison of atomic charges on the boron atoms across all three complexes shows minimal differences (Tables S15–S17). Therefore, we attribute this increase in An–B distances upon reduction to a push–pull effect without involvement of orbital interactions. This is consistent with the oxidation state assignment of An(III) in both the neutral and reduced species. The formation of a δ-bond does impact the An–B distances slightly, but not to the extent one would observe if a metal-centered reduction had taken place.

**Table 7:** Natural Orbital Occupation Numbers (NOONs) and Effective Bond Orders between the An(III) and Arene Ligand in the Lower-Spin States of the Reduced  Complexes

complex	spin	NOON_bonding_	NOON_antibonding_	EBO
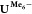	1	1.79	0.18	0.805
	2	0.94	0.09	0.835
		0.93	0.11	
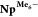	3/2	1.40	0.53	0.435
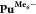	2	1.27	0.66	0.305

## Conclusions

The molecular geometries, electronic structure, and chemical bonding in complexes with soft-donor arene and borohydride ligands coordinated to uranium, neptunium, and plutonium were studied in  series. Density Functional Theory (DFT) calculations reveal a gradual increase in the average An–arene distances from U to Pu in the neutral complexes. This is contrary to the trend in the ionic radii of U, Np, and Pu ions, but consistent with a weaker bond across the series. This reduction in bond strength is associated with a decrease in the percent orbitalic contribution to the bond and a simultaneous increase in the percent of nonorbital contributions (i.e., electrostatic and dispersion forces). The 5f-orbitals are stabilized from U to Pu contributing to less favorable actinide-arene and actinide-borohydride interactions. This is consistent with the reduction in metal-arene orbital mixing in the complete active space (CASSCF) natural orbitals. DFT-derived analyses (EDA, QTAIM, and delocalization indices) show that in the neutral complexes, the An–arene and An–borohydride interactions are primarily electrostatic. Additionally, the strength of both metal–ligand interactions decreases. In the actinide-arene interaction, this is due to a reduction in both orbital- and degeneracy-driven covalency; however, in the actinide-borohydride interaction, this is attributed to a reduction in degeneracy-driven covalency only.

Upon reduction to form , the An–arene distances contract by approximately 0.1–0.2 Å, due to the formation of δ-bonding interactions resulting in stronger metal–ligand interactions compared to the neutral complexes. These δ-orbitals have contributions from the 5f- and 6d-orbitals of the metal ion as well as the π*-orbitals of arene. The extent of 6d-orbital mixing decreases from U to Np, and no longer has any contribution in Pu. This manifests in the effective bond order (EBO) which decreases from 0.835 in U to 0.305 in Pu due to an increase in the population of the δ*-antibonding orbital, as well as an increase in the An–arene distances. Finally, the ground states are assigned for all three  complexes and can be best described as An(III) metal ions interacting with an arene radical. Ferromagnetic coupling is observed in the high-spin state, while antiferromagnetic coupling occurs in the lower-spin states. Since the arene is reduced, the metal center remains in the +3 oxidation state; therefore, the actinide-borohydride interaction is less effected by reduction compared to the actinide-arene interaction. However, the An–B distances elongate upon reduction due to changes at the An(III) center induced by δ-bond formation. CASPT2 computed electron affinities show that the reduction becomes less favorable moving from U to Pu. Therefore, not only does covalency decrease across the series, but the stability of the complexes also diminishes in this particular ligand framework. This suggests that alternative ligand platforms should be explored, particularly to stabilize a terminal Pu–arene bond.

## Data Availability

The input and output files associated with all calculations are also available in a FigShare repository DOI: 10.6084/m9.figshare.28195664.

## Supporting Information Available

To ensure reproducibility, XYZ coordinates are provided (ZIP)
Active orbital images, topology analysis, delocalization index, spin–orbit coupling, electron affinity, and assignment of oxidation states to the metal ions in the reduced complexes are included (PDF)
