# Supplementary material for: Computational Investigation of the Chemical Bond between An(III) Ions and Soft-Donor Ligands
Source: Inorg Chem. 2025 Mar 21;64(12):5866–77. doi: 10.1021/acs.inorgchem.4c03924 (PMC11962836; doi:10.1021/acs.inorgchem.4c03924)
Supplement: Supplementary file 2 — ic4c03924_si_002.pdf [file ic4c03924_si_002.pdf]

Supporting Information for: Computational Investigation of the Chemical Bond Between An(III) Ions and Soft-Donor Ligands

Sabyasachi Roy Chowdhury,*,†,‡ Naomi Rehberg,‡ and Bess Vlaisavljevich†,‡

†*Department of Chemistry, University of Iowa, Iowa City, Iowa, 52242, USA*

‡*Department of Chemistry, University of South Dakota, Vermillion, South Dakota, 57069, USA*

E-mail: sabyasachi-roychowdhury@uiowa.edu

List of Tables

S1 Comparison of the average U–Arene, U–B, and U–H distances (in Å) of the $\mathbf{U^{Me_6}}$ and $\mathbf{U^{Me_6^-}}$ geometries obtained from the gas and solvent phase calculations. The COSMO solvation model with toluene was used as a solvent.[1] S12

S2 Comparison of CASPT2 energies (in a.u.) calculated on the DFT optimized $\mathbf{U^{Me_6}}$ and $\mathbf{U^{Me_6^-}}$ geometries in both the gas phase and implicit solvation using the COSMO solvation model,[1] with toluene as the solvent. An active space of (9e,18o) was used for the neutral complexes and the reduced systems was described by (10e,18o) active space. S12

S3 Comparison of the PBE0 computed Kohn Sham orbital energies (α spin) for the An^{Me_6} complexes obtained from the geometry optimization. The An 5f-orbitals are occupied, whereas the δ- and π^*-orbitals are empty. All the orbital energies are in eV. For $[U^{Me_6}]_{Solv}$, COSMO solvation model was used with toluene as solvent.[1] S13

S4 The average topological properties of $An-C_{arene}$ bonds computed at the bond critical points (BCPs). All values are expressed in atomic units.The topology metrics of U^{Me_6} are taken from *Chem. Sci., 2024,15, 1810-1819*. S15

S5 The average topological properties of An−B bonds of the complexes, computed at the bond critical points (BCPs). All values are expressed in atomic units. The topology metrics of U^{Me_6} are taken from *Chem. Sci., 2024,15, 1810-1819*. S15

S6 Delocalization indices (δ) between the metal ions and arene carbon atoms of the the complexes. The δ of U^{Me_6} are taken from *Chem. Sci., 2024,15, 1810-1819*. . . . S15

S7 Delocalization indices (δ) between the metal ions and boron atoms of the complexes. The δ of U^{Me_6} are taken from *Chem. Sci., 2024,15, 1810-1819*. S15

S8 Delocalization indices (δ) between the metal ions and the three hydrogens of borohydrides directed towards the metal ions. S16

S9 Selected geometric parameters (PBE0) of the $\mathbf{An^{Me_6}}^-$ complexes in different spin states. The deviation of bond distances from the neutral geometries are provided in parentheses. Distances are in Åand angles in degrees. The geometric parameters of the S=2 and S=1 spin states of $U^{Me_6^-}$ are taken from *Chem. Sci., 2024,15, 1810-1819*.S16

S10 The lowest CASPT2 and spin-orbit-CASPT2 (SO-CASPT2) energy levels of the neutral $\mathbf{U^{Me_6}}$ and reduced $\mathbf{U^{Me_6}}^-$ complexes. The PBE0 geometry used is noted in the column labeled “Complex”. S18

S11 The lowest CASPT2 and spin-orbit-CASPT2 (SO-CASPT2) energy levels of the neutral $\mathbf{Np^{Me_6}}$ and reduced $\mathbf{Np^{Me_6}}^-$ complexes. The PBE0 geometry used is noted in the column labeled “Complex”. S19

S12 The lowest CASPT2 and spin-orbit-CASPT2 (SO-CASPT2) energy levels of the neutral $\mathbf{Pu^{Me_6}}$ and reduced $\mathbf{Pu^{Me_6^-}}$ complexes. The PBE0 geometry used is noted in the column labeled “Complex”. The subscripts 1, 2 and 3 in the composition column denote the first, second and third S=5/2 CASPT2 states. S20

S13 Comparison of the lowest SO-CASPT2 energies (in a.u.) of the **Pu**$^{\mathbf{Me_6^-}}$ complexes, computed on the DFT optimized S=3 and S=2 geometries S22
S14 DFT and CASPT2 computed electron affinities of the **An**$^{\mathbf{Me_6}}$/**An**$^{\mathbf{Me_6}-}$ complexes. All energies are in kcal/mol. S23
S15 LoProp charges on each of the B-atoms, computed from CASSCF-(9*e*,18*o*) calculations, for the quartet state of **U**$^{\mathbf{Me_6}}$, and with the (10*e*,18*o*) active space for the quintet and triplet state of [**U**$^{\mathbf{Me_6}}$]$^-$. S24
S16 LoProp charges on each of the B-atoms, computed from CASSCF-(10*e*,18*o*) calculations, for the quintet state of **Np**$^{\mathbf{Me_6}}$, and with the (11*e*,18*o*) active space for the sextet and quartet state of [**Np**$^{\mathbf{Me_6}}$]$^-$. S24
S17 LoProp charges on each of the B-atoms, computed from CASSCF-(11*e*,18*o*) calculations, for the sextet state of **Pu**$^{\mathbf{Me_6}}$, and with the (12*e*,18*o*) active space for the septet and quintet state of [**Pu**$^{\mathbf{Me_6}}$]$^-$. S24
S18 LoProp charge analysis from CASSCF-(10*e*,18*o*) for the quintet state of **Np**$^{\mathbf{Me_6}}$, and with (11*e*,18*o*) active space for the sextet and quartet state of [**Np**$^{\mathbf{Me_6}}$]$^-$. S25
S19 Mulliken spin population analysis from CASSCF-(10*e*,18*o*) for the quintet state of **Np**$^{\mathbf{Me_6}}$, and with (11*e*,18*o*) active space for the sextet and quartet state of [**Np**$^{\mathbf{Me_6}}$]$^-$. S25
S20 LoProp charge analysis from CASSCF-(11*e*,18*o*) for the sextet state of **Pu**$^{\mathbf{Me_6}}$, and with (12*e*,18*o*) active space for the septet and quintet state of [**Pu**$^{\mathbf{Me_6}}$]$^-$. S25
S21 Mulliken spin population analysis from CASSCF-(11*e*,18*o*) for the sextet state of **Pu**$^{\mathbf{Me_6}}$, and with (12*e*,18*o*) active space for the septet and quintet state of [**Pu**$^{\mathbf{Me_6}}$]$^-$. S25

List of Figures

S1 The CASSCF active natural orbitals of the **U**$^{\mathbf{Me_6}}$ complex from the (9*e*, 13*o*) active space. An isosurface value of 0.04 a.u. was used. Occupation numbers and atomic contributions to the orbitals are included. S6

S2 The CASSCF active natural orbitals of the $\mathbf{Np}^{\mathbf{Me_6}}$ complex from the (10*e*, 13*o*) active space. An isosurface value of 0.04 a.u. was used. Occupation numbers and atomic contributions to the orbitals are included. S7
S3 The CASSCF active natural orbitals of the $\mathbf{Pu}^{\mathbf{Me_6}}$ complex from the (11*e*, 13*o*) active space. An isosurface value of 0.04 a.u. was used. Occupation numbers and atomic contributions to the orbitals are included. S7
S4 The CASSCF active natural orbitals of the $\mathbf{U}^{\mathbf{Me_6^-}}$, S=2 complex from the (10*e*, 18*o*) active space. An isosurface value of 0.04 a.u. was used. Occupation numbers and atomic contributions to the orbitals are included. S8
S5 The CASSCF active natural orbitals of the $\mathbf{U}^{\mathbf{Me_6^-}}$, S=1 complex from the (10*e*, 18*o*) active space. An isosurface value of 0.04 a.u. was used. Occupation numbers and atomic contributions to the orbitals are included. S9
S6 The CASSCF active natural orbitals of the $\mathbf{Np}^{\mathbf{Me_6^-}}$, S=5/2 complex from the (11*e*, 18*o*) active space. An isosurface value of 0.04 a.u. was used. Occupation numbers and atomic contributions to the orbitals are included. S10
S7 The CASSCF active natural orbitals of the $\mathbf{Np}^{\mathbf{Me_6^-}}$, S=3/2 complex from the (11*e*, 18*o*) active space. An isosurface value of 0.04 a.u. was used. Occupation numbers and atomic contributions to the orbitals are included. S10
S8 The CASSCF active natural orbitals of the $\mathbf{Pu}^{\mathbf{Me_6^-}}$, S=3 complex from the (12*e*, 18*o*) active space. An isosurface value of 0.04 a.u. was used. Occupation numbers and atomic contributions to the orbitals are included. S11
S9 The CASSCF active natural orbitals of the $\mathbf{Pu}^{\mathbf{Me_6^-}}$, S=2 complex from the (12*e*, 18*o*) active space. An isosurface value of 0.04 a.u. was used. Occupation numbers and atomic contributions to the orbitals are included. S11

S10 Comparison of the DFT Kohn Sham orbital energies (α spin) of the complexes, obtained from geometry optimizations. The An $5f$-orbitals are occupied, whereas the δ- and $\pi*$-orbitals are empty. Arene orbitals are shown in red, orbitals with a $\delta-$interaction are in blue, and An $5f$-orbitals in black. S13

1. CASSCF Natural Orbitals

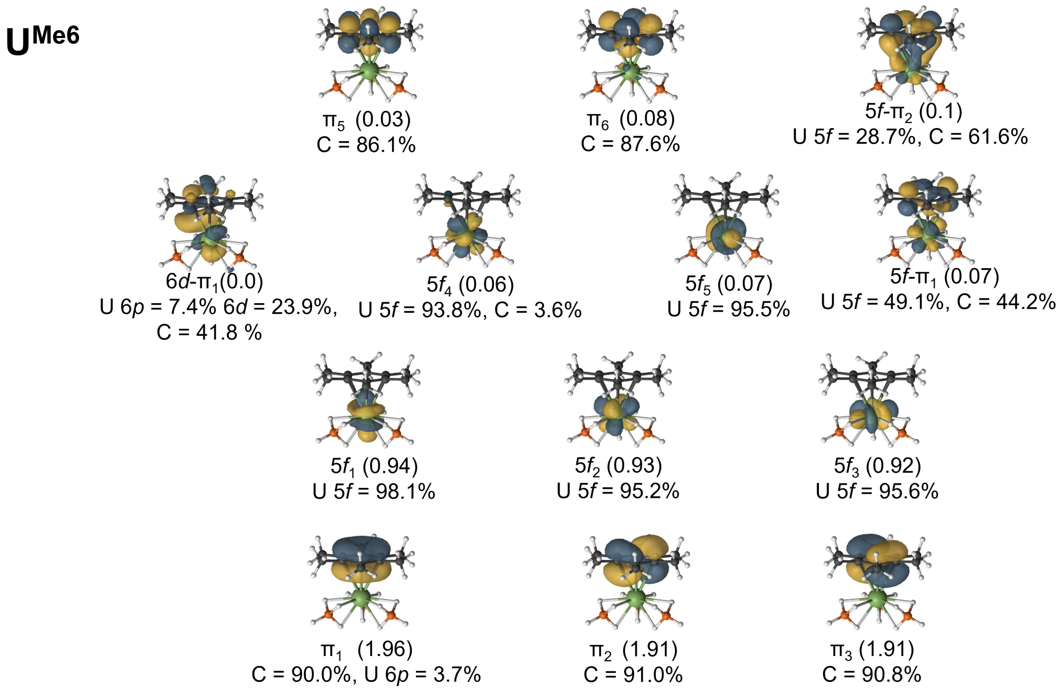

Figure S1: The CASSCF active natural orbitals of the $\mathbf{U}^{\mathbf{Me_6}}$ complex from the (9*e*, 13*o*) active space. An isosurface value of 0.04 a.u. was used. Occupation numbers and atomic contributions to the orbitals are included.

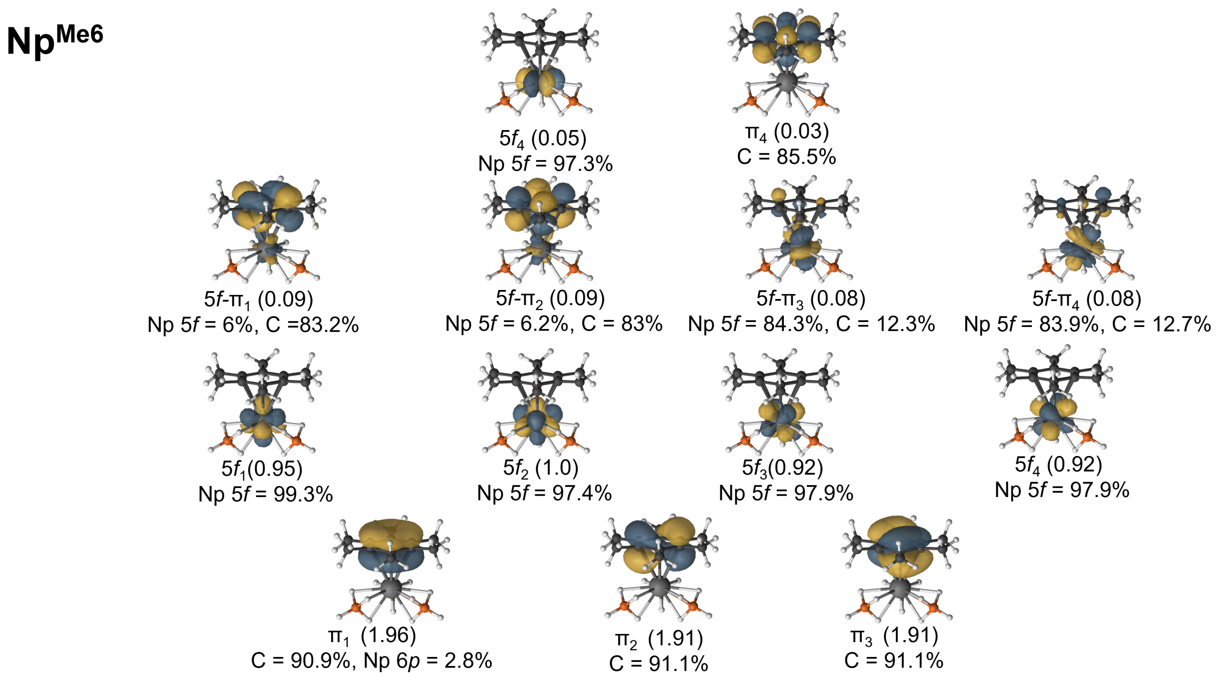

Figure S2: The CASSCF active natural orbitals of the $\mathbf{Np^{Me_6}}$ complex from the (10*e*, 13*o*) active space. An isosurface value of 0.04 a.u. was used. Occupation numbers and atomic contributions to the orbitals are included.

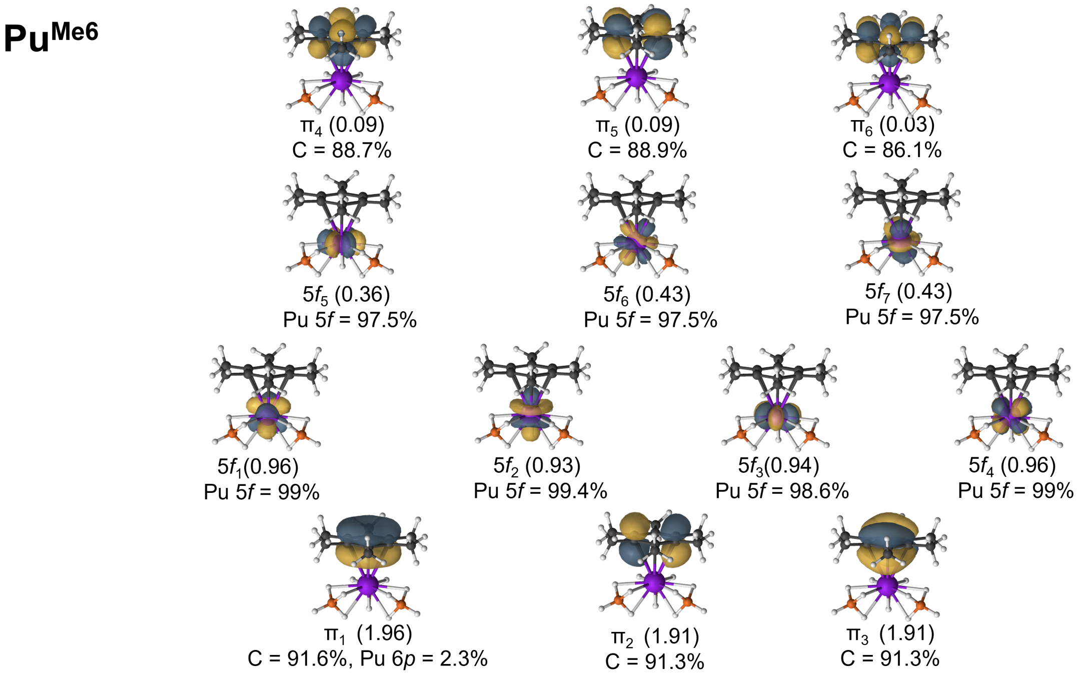

Figure S3: The CASSCF active natural orbitals of the $\mathbf{Pu^{Me_6}}$ complex from the (11*e*, 13*o*) active space. An isosurface value of 0.04 a.u. was used. Occupation numbers and atomic contributions to the orbitals are included.

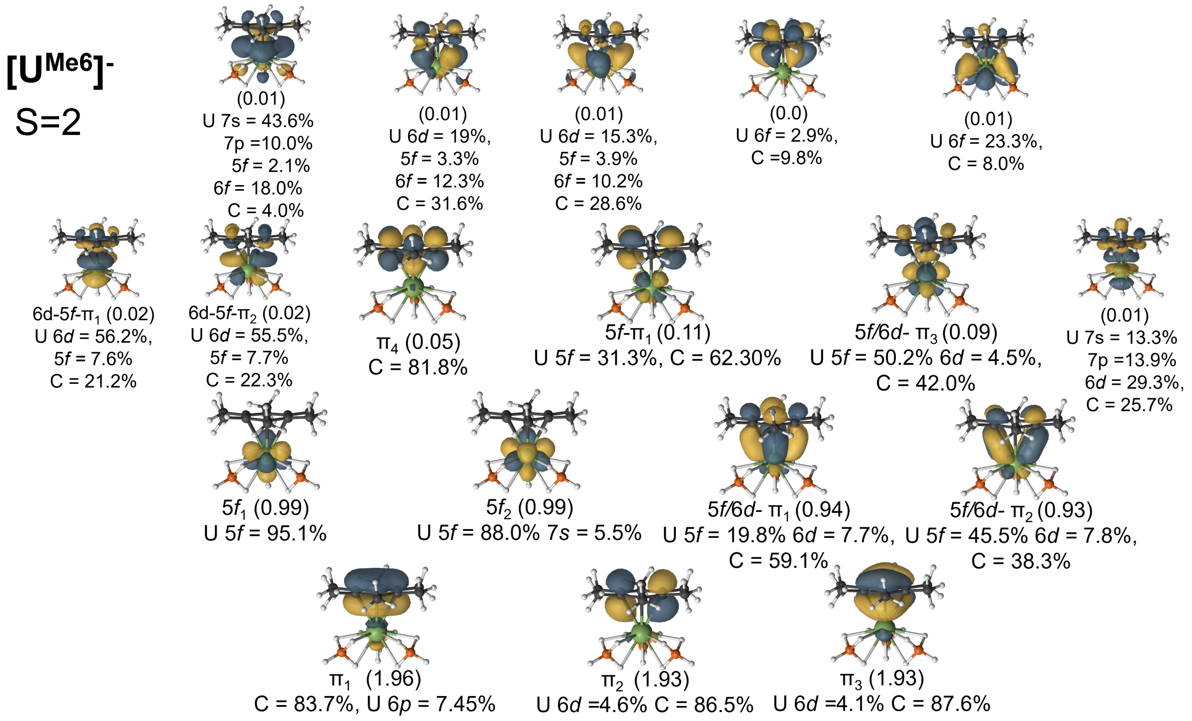

Figure S4: The CASSCF active natural orbitals of the $\mathbf{U^{Me_6^-}}$, S=2 complex from the (10*e*, 18*o*) active space. An isosurface value of 0.04 a.u. was used. Occupation numbers and atomic contributions to the orbitals are included.

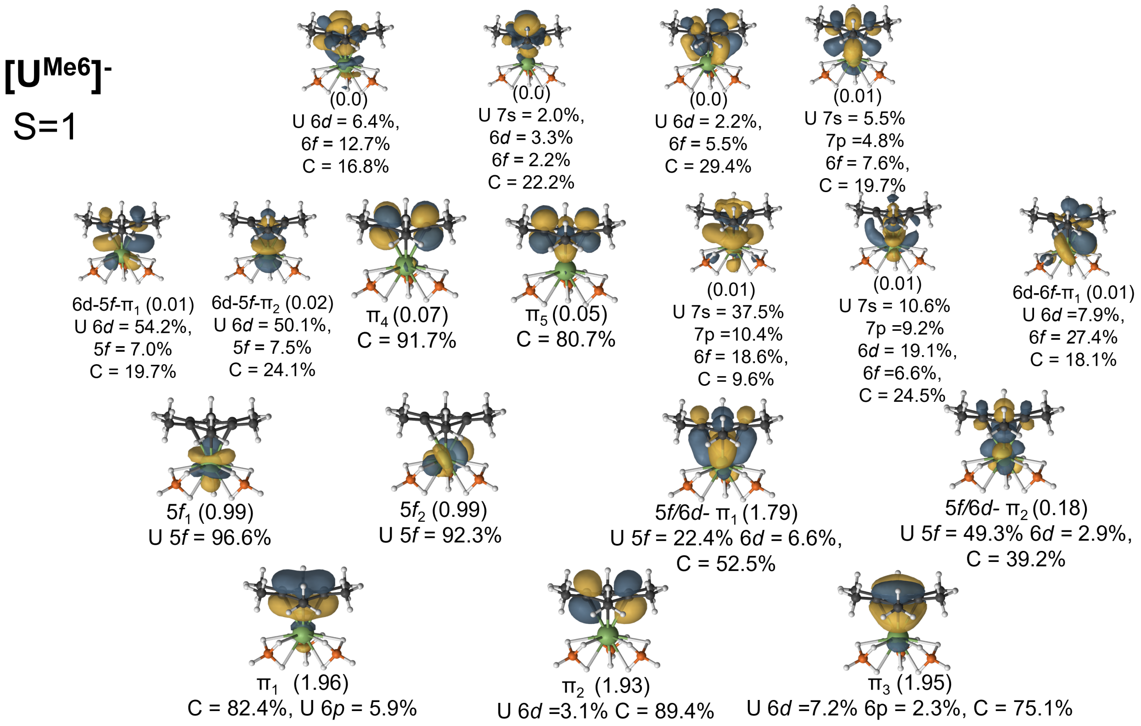

Figure S5: The CASSCF active natural orbitals of the $\mathbf{U^{Me_6^-}}$, S=1 complex from the (10*e*, 18*o*) active space. An isosurface value of 0.04 a.u. was used. Occupation numbers and atomic contributions to the orbitals are included.

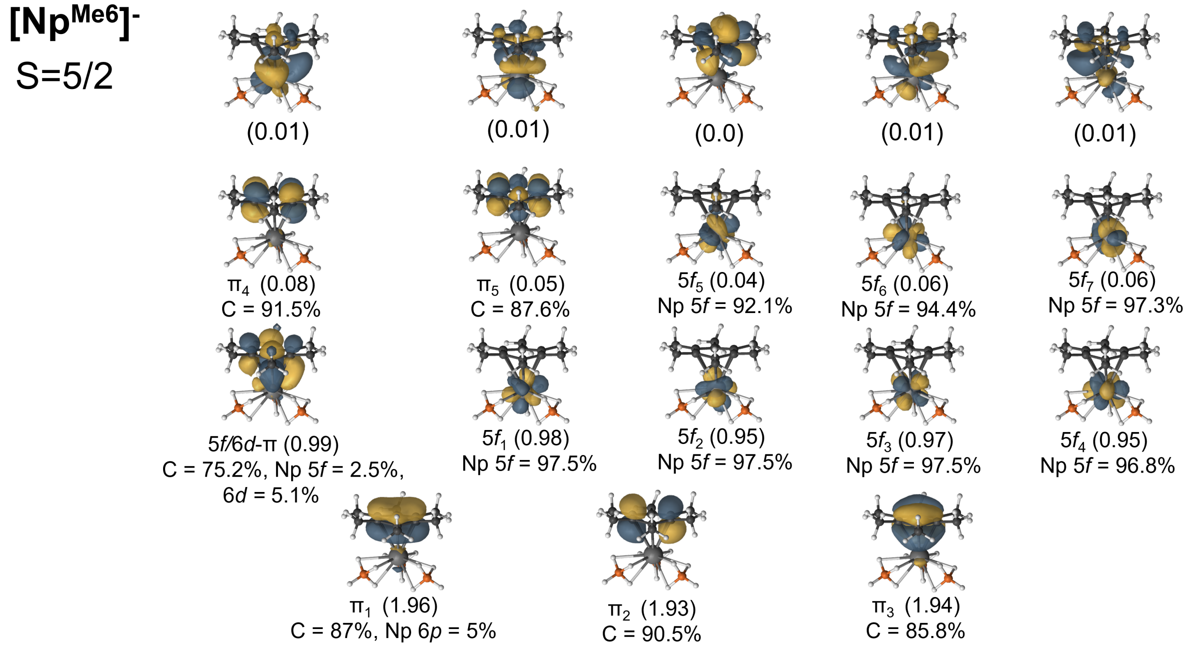

Figure S6: The CASSCF active natural orbitals of the $\mathbf{Np^{Me_6^-}}$, S=5/2 complex from the (11*e*, 18*o*) active space. An isosurface value of 0.04 a.u. was used. Occupation numbers and atomic contributions to the orbitals are included.

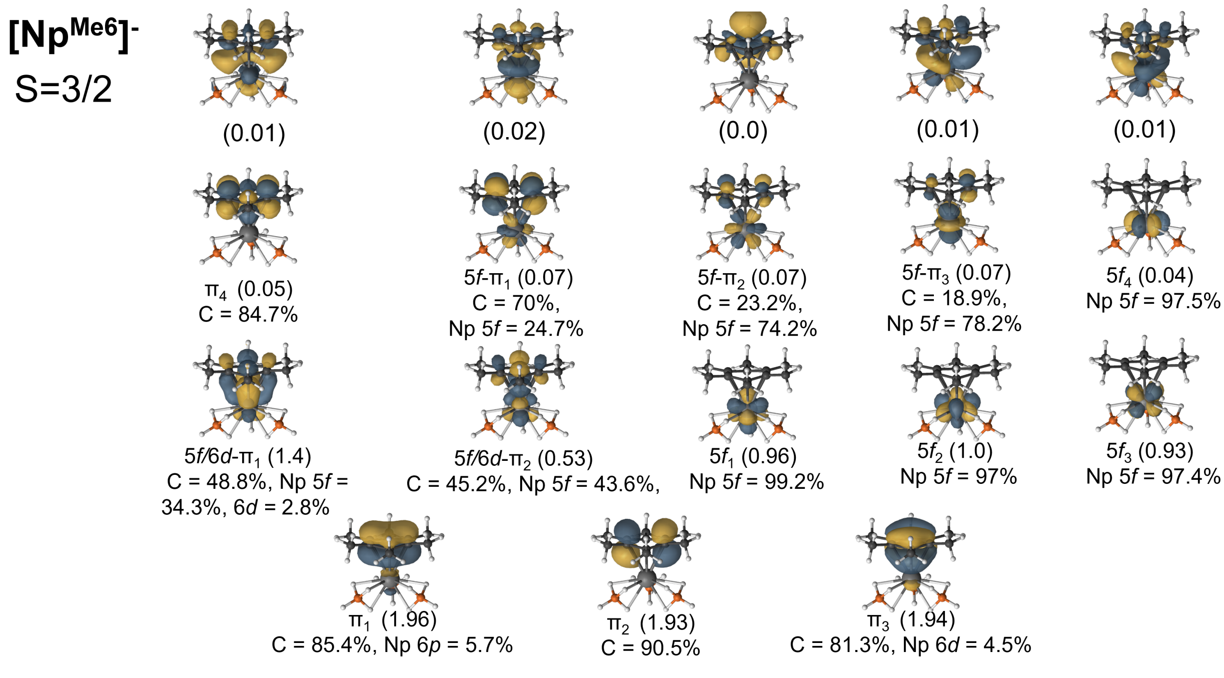

Figure S7: The CASSCF active natural orbitals of the $\mathbf{Np^{Me_6^-}}$, S=3/2 complex from the (11*e*, 18*o*) active space. An isosurface value of 0.04 a.u. was used. Occupation numbers and atomic contributions to the orbitals are included.

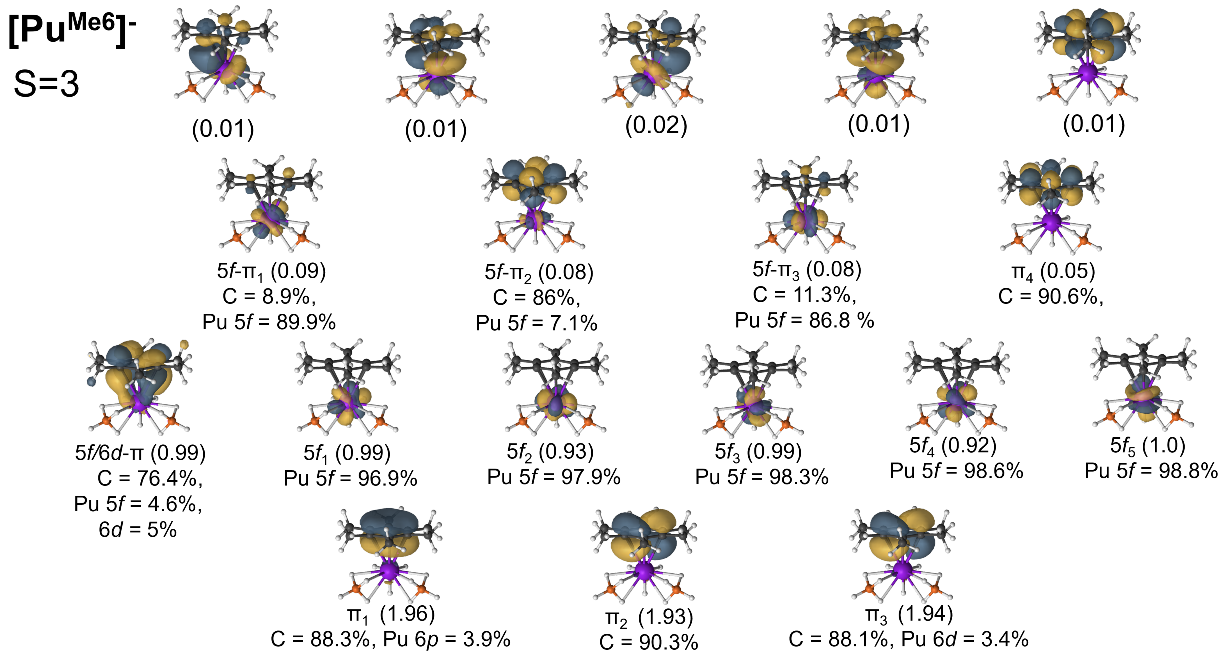

Figure S8: The CASSCF active natural orbitals of the **Pu**$^{\mathbf{Me}_6^-}$, S=3 complex from the (12*e*, 18*o*) active space. An isosurface value of 0.04 a.u. was used. Occupation numbers and atomic contributions to the orbitals are included.

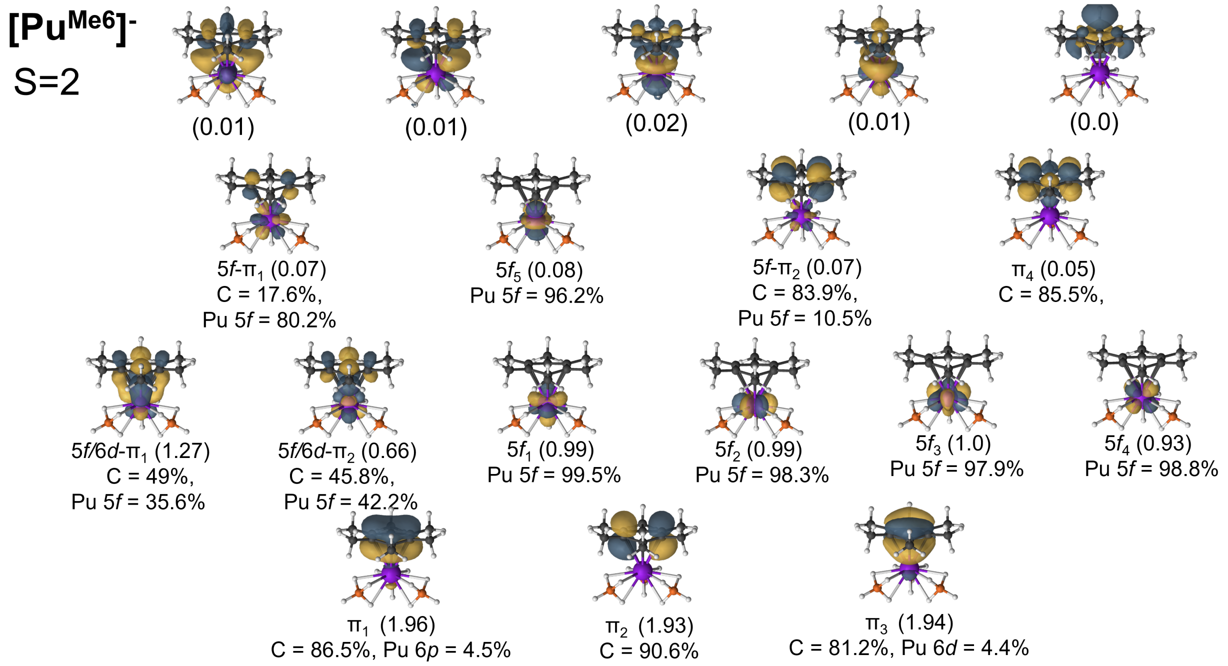

Figure S9: The CASSCF active natural orbitals of the **Pu**$^{\mathbf{Me}_6^-}$, S=2 complex from the (12*e*, 18*o*) active space. An isosurface value of 0.04 a.u. was used. Occupation numbers and atomic contributions to the orbitals are included.

2. DFT Analysis

Table S1: Comparison of the average U–Arene, U–B, and U–H distances (in Å) of the $\mathbf{U^{Me_6}}$ and $\mathbf{U^{Me_6^-}}$ geometries obtained from the gas and solvent phase calculations. The COSMO solvation model with toluene was used as a solvent.[1]

	Bond	$\mathbf{U^{Me_6}}$	$\mathbf{U^{Me_6^-}}$, S=2	$\mathbf{U^{Me_6^-}}$, S=1
Gas Phase	An–Arene	2.903	2.709	2.703
Toluene	An–Arene	2.901	2.693	2.696
Gas Phase	An–B	2.525	2.593	2.587
Toluene	An–B	2.531	2.600	2.594
Gas Phase	An–H	2.337	2.412	2.404
Toluene	An–H	2.346	2.420	2.414

Table S2: Comparison of CASPT2 energies (in a.u.) calculated on the DFT optimized $\mathbf{U^{Me_6}}$ and $\mathbf{U^{Me_6^-}}$ geometries in both the gas phase and implicit solvation using the COSMO solvation model,[1] with toluene as the solvent. An active space of (9*e*,18*o*) was used for the neutral complexes and the reduced systems was described by (10*e*,18*o*) active space.

	$\mathbf{U^{Me_6}}$	$\mathbf{U^{Me_6^-}}$, S=2	$\mathbf{U^{Me_6^-}}$, S=1
Gas Phase	-28498.6013663125	-28498.6465058364	-28498.6424500049
Toluene	-28498.6021982201	-28498.6469023362	-28498.6417975126
Difference (kcal/mol)	0.5	0.2	0.4

Table S3: Comparison of the PBE0 computed Kohn Sham orbital energies (α spin) for the An^{Me_6} complexes obtained from the geometry optimization. The An 5f-orbitals are occupied, whereas the δ- and π^*-orbitals are empty. All the orbital energies are in eV. For $[U^{Me_6}]_{Solv}$, COSMO solvation model was used with toluene as solvent.[1]

Orbitals	U^{Me_6}	$[U^{Me_6}]_{Solv}$	Np^{Me_6}	Pu^{Me_6}
Arene (π^*)	-1.390	-1.310	-1.401	-1.325
Arene (π^*)	-1.394	-1.314	-1.402	-1.332
δ ($5f/6d$-π)	-1.570	-1.482	-1.556	-1.497
δ ($5f/6d$-π)	-1.572	-1.483	-1.556	-1.526
An ($5f$)	-4.974	-5.020	-5.781	-6.114
	-4.974	-5.020	-5.782	-6.790
	-5.255	-5.284	-5.918	-6.833
			-6.304	-7.268
				-7.377

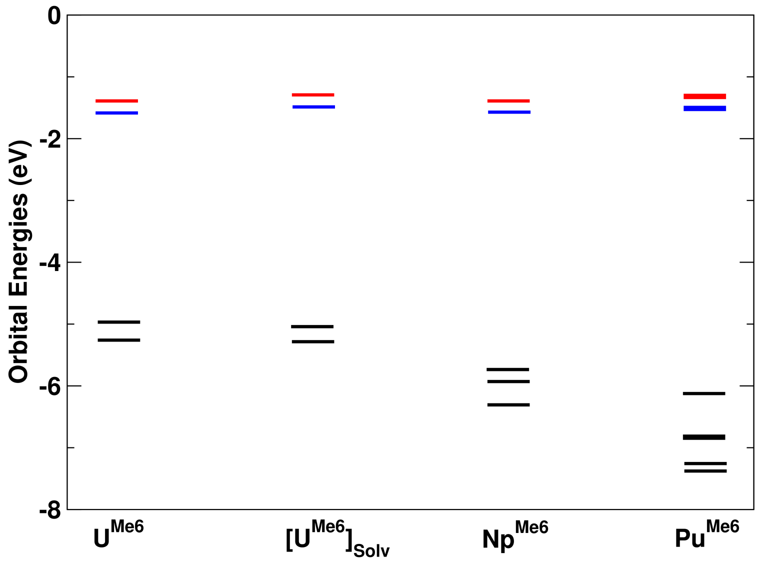

Figure S10: Comparison of the DFT Kohn Sham orbital energies (α spin) of the complexes, obtained from geometry optimizations. The An 5f-orbitals are occupied, whereas the δ- and $\pi*$-orbitals are empty. Arene orbitals are shown in red, orbitals with a δ−interaction are in blue, and An 5f-orbitals in black.

Topology Analysis

To understand the chemical bonding between uranium and arene moieties, we used the quantum theory of atoms in molecules (QTAIM) developed by Bader.[2] According to this theory, a chemical bond exists if there is a line of locally maximum electron density that links two neighboring atoms and a bond critical point (BCP) is identified. A BCP is defined as a minimum in the density along the locally maximal line. At a BCP, the gradient of the electron density ($\nabla\rho$) is zero, and the Laplacian ($\nabla^2\rho$) has either a net positive or net negative value. In the most clear cases, a positive Laplacian means a local depletion of charge, consistent with an ionic bond. On the other hand, a negative Laplacian indicates a local concentration of charge, which is a strong indication of a covalent bond. However, if the Laplacian is slightly positive, the total electronic energy density, E(r), which is the sum total of the kinetic and potential energy densities at the BCP, can be used to further classify the bond.[3] In a perfectly covalent bond, both the $\nabla^2\rho$ and E(r) are negative. A positive $\nabla^2\rho$ and a negative E(r) indicates a dative bond, a zero or close to zero E(r) indicates a metallic bond, and a positive E(r) indicates either an ionic or van der Waals bond. In all the systems we find positive $\nabla^2\rho$ and negative E(r) supporting the assignment of uranium-arene bonds as dative bonds. The topology analysis was performed using Multiwfn 3.8 program.[4]

Delocalization Index

The delocalization indices (δ) between the metal ion and the ligands were computed using the basin analysis approach as implemented in the Multiwfn 3.8 program.[4] This metric has been used in actinide chemistry to support the presence of degeneracy-driven covalency, but can also increase with contributions from orbital-driven covalency. In these systems, since ρ remains constant across the serins in the QTAIM analysis, contributions from orbital-effects are not expected to dominate.

Table S4: The average topological properties of An − C_{arene} bonds computed at the bond critical points (BCPs). All values are expressed in atomic units.The topology metrics of U^{Me_6} are taken from *Chem. Sci., 2024,15, 1810-1819*.

Complex	ρ	$\nabla^2\rho$	G(r)	V(r)	E(r)
U^{Me_6}	0.0300	0.0744	0.0212	-0.0238	-0.0026
Np^{Me_6}	0.0271	0.0761	0.0207	-0.0224	-0.0017
Pu^{Me_6}	0.0244	0.0700	0.0186	-0.0197	-0.0011

Table S5: The average topological properties of An−B bonds of the complexes, computed at the bond critical points (BCPs). All values are expressed in atomic units. The topology metrics of U^{Me_6} are taken from *Chem. Sci., 2024,15, 1810-1819*.

Complex	ρ	$\nabla^2\rho$	G(r)	V(r)	E(r)
U^{Me_6}	0.0543	0.128	0.0451	-0.0582	-0.0132
Np^{Me_6}	0.0539	0.1333	0.0461	-0.0589	-0.0128
Pu^{Me_6}	0.0520	0.1352	0.0454	-0.0571	-0.0117

Table S6: Delocalization indices (δ) between the metal ions and arene carbon atoms of the the complexes. The δ of U^{Me_6} are taken from *Chem. Sci., 2024,15, 1810-1819*.

Complex (Å)	An-C1	An-C2	An-C3	An-C4	An-C5	An-C6	Average
U^{Me_6}	0.158	0.159	0.153	0.163	0.152	0.159	0.157
Np^{Me_6}	0.125	0.139	0.127	0.136	0.126	0.135	0.131
Pu^{Me_6}	0.098	0.114	0.102	0.110	0.108	0.110	0.107

Table S7: Delocalization indices (δ) between the metal ions and boron atoms of the complexes. The δ of U^{Me_6} are taken from *Chem. Sci., 2024,15, 1810-1819*.

Complex (Å)	An-B1	An-B2	An-B3	Average
U^{Me_6}	0.158	0.158	0.158	0.158
Np^{Me_6}	0.155	0.153	0.154	0.154
Pu^{Me_6}	0.146	0.139	0.146	0.144

Table S8: Delocalization indices (δ) between the metal ions and the three hydrogens of borohydrides directed towards the metal ions.

An-H	U^{Me_6}	Np^{Me_6}	Pu^{Me_6}
An-H1	0.232	0.233	0.231
An-H2	0.240	0.235	0.217
An-H3	0.230	0.231	0.209
An-H4	0.229	0.230	0.227
An-H5	0.238	0.236	0.214
An-H6	0.233	0.231	0.212
An-H7	0.231	0.231	0.218
An-H8	0.239	0.235	0.234
An-H9	0.230	0.230	0.216
Avg.	0.234	0.232	0.220

Table S9: Selected geometric parameters (PBE0) of the $\mathbf{An^{Me_6}}^-$ complexes in different spin states. The deviation of bond distances from the neutral geometries are provided in parentheses. Distances are in Åand angles in degrees. The geometric parameters of the S=2 and S=1 spin states of $U^{Me_6^-}$ are taken from *Chem. Sci., 2024,15, 1810-1819*.

Complex	Spin	An−arene	An−B
$\mathbf{U^{Me_6}}^-$	2	2.709 (-0.194)	2.593 (0.068)
	1	2.703 (-0.200)	2.587 (0.062)
	0	2.563 (-0.34)	2.573 (0.048)
$\mathbf{Np^{Me_6}}^-$	5/2	2.818 (-0.110)	2.600 (0.090)
	3/2	2.759 (-0.169)	2.572 (0.062)
	1/2	2.730 (-0.198)	2.563 (0.053)
$\mathbf{Pu^{Me_6}}^-$	3	2.870 (-0.106)	2.634 (0.121)
	2	2.781 (-0.195)	2.580 (0.067)
	1	2.753 (-0.223)	2.566 (0.053)
	0	2.708 (-0.268)	2.531 (0.018)

3. Spin-Orbit Coupling

The scalar-relativistic states obtained from (XMS)-CASPT2 calculations were subjected to a state-interaction Hamiltonian as implemented in the RASSI module of OpenMolcas to evaluate the spin-orbit mixing of the states *a posteriori*.[5] For the neutral $\mathbf{U}^{\mathbf{Me_6}}$ complex, the CASPT2 computed lowest S=3/2 and S=1/2 states were employed as the N-electron basis to evaluate the spin–orbit mixing of the so-called spin-free states (Table S10). Similarly, the S=2, S=1, and S=0 states were used for $\mathbf{Np}^{\mathbf{Me_6}}$ (Table S11). In the case of $\mathbf{Pu}^{\mathbf{Me_6}}$, the S=5/2 state was three-fold degenerate; therefore, three S=5/2 states were computed with XMS-CASPT2, along with the lowest-energy S=3/2 and S=1/2 states. These spin-free states were used in the state-interaction Hamiltonian to predict their mixing upon including spin-orbit coupling (Table S12).

For the reduced $[\mathbf{An}^{\mathbf{Me_6}}]^-$ complexes, the CASPT2 computed lowest-energy spin-multiplets were subjected to spin-orbit coupling, irrespective of the spin-state associated with the molecular geometries. Specifically, the lowest energy S=2, S=1 and S=0 states were employed for $[\mathbf{U}^{\mathbf{Me_6}}]^-$, while the S=5/2, S=3/2 and S=1/2 states were used for the $[\mathbf{Np}^{\mathbf{Me_6}}]^-$. For $[\mathbf{Pu}^{\mathbf{Me_6}}]^-$, the S=3, S=2, S=1 and S=0 states were considered to evaluate spin-orbit coupling. The lowest spin-free energy levels, the spin-orbit states generated from these spin-free states, and the composition of the spin-orbit states are summarized in Tables S10 to S12. The spin-orbit coupling calculations were carried out using OpenMolcas software package.[6]

Table S10: The lowest CASPT2 and spin-orbit-CASPT2 (SO-CASPT2) energy levels of the neutral $\mathbf{U}^{\mathbf{Me_6}}$ and reduced $\mathbf{U}^{\mathbf{Me_6}-}$ complexes. The PBE0 geometry used is noted in the column labeled "Complex".

Complex	Spin	CASPT2 energies (cm^{-1})	SO-CASPT2 energies (cm^{-1})	Composition of SO-CASPT2 States
$\mathbf{U}^{\mathbf{Me_6}}$	3/2	0.0	0.0	99.4% $\|3/2\rangle$
S=3/2	1/2	7178	0.0	99.4% $\|3/2\rangle$
			44.5	100% $\|3/2\rangle$
			44.5	100% $\|3/2\rangle$
			7267.1	99.4% $\|1/2\rangle$
			7267.1	99.4% $\|1/2\rangle$
$\mathbf{U}^{\mathbf{Me_6}-}$	2	0.0	0.0	92% $\|2\rangle$ + 8% $\|1\rangle$
S=2	1	2778.5	60.5	93% $\|2\rangle$ + 7% $\|1\rangle$
	0	7804.5	62.6	94% $\|2\rangle$ + 6% $\|1\rangle$
			267.1	100% $\|2\rangle$
			267.1	100% $\|2\rangle$
			3198.4	92% $\|1\rangle$ + 7% $\|2\rangle$
			3250.0	94% $\|1\rangle$ + 6% $\|2\rangle$
			3312.1	92% $\|1\rangle$ + 8% $\|2\rangle$
			8125.9	99% $\|0\rangle$
$\mathbf{U}^{\mathbf{Me_6}-}$	2	2812.7	0.0	93% $\|1\rangle$ + 6% $\|2\rangle$
S=1	1	0.0	55.9	92% $\|1\rangle$ + 8% $\|2\rangle$
	0	5546.3	115.2	94% $\|1\rangle$ + 6% $\|2\rangle$
			3121.1	100 % $\|2\rangle$
			3121.1	100 % $\|2\rangle$
			3298.7	94% $\|2\rangle$ + 5% $\|1\rangle$
			3314.3	94% $\|2\rangle$ + 6% $\|1\rangle$
			3373.7	92% $\|2\rangle$ + 8% $\|1\rangle$
			5985.5	98% $\|0\rangle$ + 2% $\|1\rangle$

Table S11: The lowest CASPT2 and spin-orbit-CASPT2 (SO-CASPT2) energy levels of the neutral **Np^{Me_6}** and reduced **Np$^{Me_6 -}$** complexes. The PBE0 geometry used is noted in the column labeled "Complex".

Complex	Spin	CASPT2 energies (cm^{-1})	SO-CASPT2 energies (cm^{-1})	Composition of SO-CASPT2 States
**Np^{Me_6}**	2	0.0	0.0	100% $\|2\rangle$
S=2	1	11336.5	8.0	100% $\|2\rangle$
	0	16781.2	8.0	100% $\|2\rangle$
			32.2	100% $\|2\rangle$
			32.2	100% $\|2\rangle$
			11389.3	100% $\|1\rangle$
			11392.8	100% $\|1\rangle$
			11400.8	100% $\|1\rangle$
			16816.9	100% $\|0\rangle$
**Np$^{Me_6 -}$**	5/2	1565.9	0.0	87% $\|3/2\rangle$ + 13% $\|5/2\rangle$
S=5/2	3/2	0.0	0.0	87% $\|3/2\rangle$ + 13% $\|5/2\rangle$
	1/2	11077.4	85.1	90% $\|3/2\rangle$ + 10% $\|5/2\rangle$
			85.1	90% $\|3/2\rangle$ + 10% $\|5/2\rangle$
			1847.5	100% $\|5/2\rangle$
			1847.5	100% $\|5/2\rangle$
			2040.5	90% $\|5/2\rangle$ + 10% $\|3/2\rangle$
			2040.5	90% $\|5/2\rangle$ + 10%$\|3/2\rangle$
			2123.7	87% $\|5/2\rangle$ + 13% $\|3/2\rangle$
			2123.7	87% $\|5/2\rangle$ + 13% $\|3/2\rangle$
			11367.9	100% $\|1/2\rangle$
			11367.9	100% $\|1/2\rangle$
**Np$^{Me_6 -}$**	5/2	3644.0	0.0	97% $\|3/2\rangle$ + 3% $\|5/2\rangle$
S=3/2	3/2	0.0	0.0	97% $\|3/2\rangle$ + 3% $\|5/2\rangle$
	1/2	11081.3	40.4	98% $\|3/2\rangle$ + 2% $\|5/2\rangle$
			40.4	98% $\|3/2\rangle$ + 2% $\|5/2\rangle$
			3765.7	100% $\|5/2\rangle$
			3765.7	100% $\|5/2\rangle$
			3844.3	98% $\|5/2\rangle$ + 2% $\|3/2\rangle$
			3844.3	98% $\|5/2\rangle$ + 2% $\|3/2\rangle$
			3882.2	97% $\|5/2\rangle$ + 3% $\|3/2\rangle$
			3882.2	97% $\|5/2\rangle$ + 3% $\|3/2\rangle$
			11211.2	100% $\|1/2\rangle$
			11211.2	100% $\|1/2\rangle$

Table S12: The lowest CASPT2 and spin-orbit-CASPT2 (SO-CASPT2) energy levels of the neutral **Pu^{Me_6}** and reduced **Pu$^{Me_6^-}$** complexes. The PBE0 geometry used is noted in the column labeled "Complex". The subscripts 1, 2 and 3 in the composition column denote the first, second and third S=5/2 CASPT2 states.

Complex	Spin	CASPT2 energies (cm^{-1})	SO-CASPT2 energies (cm^{-1})	Composition of SO-CASPT2 States
**Pu^{Me_6}**	5/2	0.0	0.0	49% $\|5/2\rangle_2$ + 38% $\|5/2\rangle_1$ + 10% $\|5/2\rangle_3$ + 3% $\|3/2\rangle$
S=5/2	5/2	31.6	0.0	49% $\|5/2\rangle_2$ + 38% $\|5/2\rangle_1$ + 10% $\|5/2\rangle_3$ + 3% $\|3/2\rangle$
	5/2	1303.3	571.3	43% $\|5/2\rangle_2$ + 33% $\|5/2\rangle_1$ + 22% $\|5/2\rangle_3$ + 2% $\|3/2\rangle$
	3/2	14688.9	571.3	43% $\|5/2\rangle_2$ + 33% $\|5/2\rangle_1$ + 22% $\|5/2\rangle_3$ + 2% $\|3/2\rangle$
	1/2	21658.1	2715.2	51% $\|5/2\rangle_2$ + 45% $\|5/2\rangle_1$ + 5% $\|5/2\rangle_3$
			2715.2	51% $\|5/2\rangle_2$ + 45% $\|5/2\rangle_1$ + 5% $\|5/2\rangle_3$
			3176.6	43% $\|5/2\rangle_1$ + 30% $\|5/2\rangle_2$ + 26% $\|5/2\rangle_3$
			3176.6	43% $\|5/2\rangle_1$ + 30% $\|5/2\rangle_2$ + 26% $\|5/2\rangle_3$
			3803.4	55% $\|5/2\rangle_3$ + 26% $\|5/2\rangle_1$ + 19% $\|5/2\rangle_2$
			3803.4	55% $\|5/2\rangle_3$ + 26% $\|5/2\rangle_1$ + 19% $\|5/2\rangle_2$
			6299.8	44% $\|5/2\rangle_1$ + 41% $\|5/2\rangle_2$ + 12% $\|5/2\rangle_3$ + 3% $\|3/2\rangle$
			6299.8	44% $\|5/2\rangle_1$ + 41% $\|5/2\rangle_2$ + 12% $\|5/2\rangle_3$ + 3% $\|3/2\rangle$
			6410.9	57% $\|5/2\rangle_3$ + 25% $\|5/2\rangle_1$ + 15% $\|5/2\rangle_2$ + 2% $\|3/2\rangle$
			6410.9	57% $\|5/2\rangle_3$ + 25% $\|5/2\rangle_1$ + 15% $\|5/2\rangle_2$ + 2% $\|3/2\rangle$
			6589.7	54% $\|5/2\rangle_3$ + 24% $\|5/2\rangle_1$ + 21% $\|5/2\rangle_2$
			6589.7	54% $\|5/2\rangle_3$ + 24% $\|5/2\rangle_1$ + 21% $\|5/2\rangle_2$
			6691.3	58% $\|5/2\rangle_3$ + 22% $\|5/2\rangle_2$ + 20% $\|5/2\rangle_1$
			6691.3	58% $\|5/2\rangle_3$ + 22% $\|5/2\rangle_2$ + 20% $\|5/2\rangle_1$
			18415.6	84% $\|3/2\rangle$ + 11% $\|1/2\rangle$ + 4% $\|5/2\rangle_2$
			18415.6	84% $\|3/2\rangle$ + 11% $\|1/2\rangle$ + 4% $\|5/2\rangle_2$
			19428.7	94% $\|3/2\rangle$ + 5% $\|5/2\rangle_2$
			19428.7	94% $\|3/2\rangle$ + 5% $\|5/2\rangle_2$

			26334.4	89% |1/2⟩ + 11% |3/2⟩
			26334.4	89% |1/2⟩ + 11% |3/2⟩
**Pu**$^{Me_6-}$	3	0.0	0.0	61% |2⟩ + 37% |3⟩ + 2% |1⟩
S=3	2	167.7	45.8	58% |2⟩ + 41% |3⟩ + 1% |1⟩
	1	13159.8	107.7	57% 2 + 42% |3⟩ + 1% |1⟩
	0	21058.4	270.4	56% |3⟩ + 44% |2⟩
			275.6	56% |3⟩ + 44% |2⟩
			681.2	100% |3⟩
			681.2	100% |3⟩
			1012.3	64% |3⟩ + 35% |2⟩ + 1% |1⟩
			1033.6	60% |3⟩ + 39% |2⟩ + 1% |1⟩
			1122.1	59% |3⟩ + 40% |2⟩ + 1% |1⟩
			1287.2	57% |2⟩ + 43% |3⟩
			1293.8	57% |2⟩ + 43% |3⟩
			14090.2	95% |1⟩ + 3% |2⟩
			14202.5	97% |1⟩ + 3% |2⟩
			14278.1	97% |1⟩ + 3% |2⟩
			21894.6	98% |0⟩ + 2% |1⟩
**Pu**$^{Me_6-}$	3	1398.1	0.0	88% |2⟩ + 12% |3⟩
S=2	2	0.0	14.1	88% |2⟩ + 12% |3⟩
	1	12775.6	57.0	88% |2⟩ + 11% |3⟩
	0	21122.3	131.2	90% |2⟩ + 10% |3⟩
			136.1	90% |2⟩ + 10% |3⟩
			1733.1	100% |3⟩
			1733.1	100% |3⟩

1883.6	92% $\|3\rangle$ + 8% $\|2\rangle$
1883.8	92% $\|3\rangle$ + 8% $\|2\rangle$
1960.5	88% $\|3\rangle$ + 12% $\|2\rangle$
1971.1	87% $\|3\rangle$ + 13% $\|2\rangle$
1992.4	87% $\|3\rangle$ + 13% $\|2\rangle$
13026.1	97% $\|1\rangle$ + 2% $\|0\rangle$
13023.8	99% $\|1\rangle$
13230.8	99% $\|1\rangle$
21639.5	98% $\|0\rangle$ + 2% $\|1\rangle$

Table S13: Comparison of the lowest SO-CASPT2 energies (in a.u.) of the $\mathbf{Pu}^{\mathrm{Me}_6^-}$ complexes, computed on the DFT optimized S=3 and S=2 geometries

$\mathbf{Pu}^{\mathrm{Me}_6^-}$, S=3	$\mathbf{Pu}^{\mathrm{Me}_6^-}$, S=2	Difference (kcal/mol)
-30088.20650505	-30088.21849057	7.5

4. Electron Affinity

To evaluate the electron affinity of the complexes and their stability across different spin states, the adiabatic energy differences between anionic and neutral complexes were calculated using the same DFT functional ($\Delta E^{\mathrm{DFT}}_{\mathrm{A-N}}$) and via CASPT2 single-point calculations evaluated on the DFT geometries ($\Delta E^{\mathrm{CASPT2}}_{\mathrm{A-N}}$) (Table S14). Upon reducing $\mathbf{U}^{\mathrm{Me}_6}$ to $\mathbf{U}^{\mathrm{Me}_6}{}^-$, two nearly iso-energetic spin states are obtained. The high-spin state (S=2) undergoes stabilization by −28.3 kcal/mol (CASPT2) and −21.5 kcal/mol (DFT), while the S=1 state is stabilized by −25.8 kcal/mol (CASPT2) and −20.0 kcal/mol (DFT). Comparing the energies of $\mathbf{Np}^{\mathrm{Me}_6}$ to $\mathbf{Np}^{\mathrm{Me}_6}{}^-$, the DFT calculations indicated a marginal (2.5 kcal/mol) stability preference for the S=3/2 state over the S=5/2 complex, while CASPT2 revealed a more significant gap, with the S=3/2 state being favored by

10.4 kcal/mol (Tables 5 and S14). In the case of $\mathbf{Pu}^{\mathrm{Me_6}}$ and $\mathbf{Pu}^{\mathrm{Me_6}-}$, DFT predicted S=3 as the ground state, but CASPT2 calculations showed that the S=2 state was more stable than S=3 by 8.2 kcal/mol (Tables 5 and S14). These discrepancies between DFT and CASPT2 energies likely emerge from the increasing multiconfigurational character in lower-spin states, although we can't exclude contributions from their varying treatments of dynamical correlation.

Table S14: DFT and CASPT2 computed electron affinities of the $\mathbf{An}^{\mathrm{Me_6}}/\mathbf{An}^{\mathrm{Me_6}-}$ complexes. All energies are in kcal/mol.

Neutral Complex	Spin	Anion Complex	Spin	ΔE^{DFT}_{A-N}	ΔE^{CASPT2}_{A-N}
$\mathbf{U}^{\mathrm{Me_6}}$	3/2	$\mathbf{U}^{\mathrm{Me_6}-}$	2	-21.5	-28.3
			1	-20.0	-25.8
$\mathbf{Np}^{\mathrm{Me_6}}$	2	$\mathbf{Np}^{\mathrm{Me_6}-}$	5/2	-14.3	-11.5
			3/2	-16.8	-21.9
$\mathbf{Pu}^{\mathrm{Me_6}}$	5/2	$\mathbf{Pu}^{\mathrm{Me_6}-}$	3	-23.3	-8.2
			2	-17.2	-16.7

Nevertheless, based on the CASPT2 energies, the electron affinity for the $\mathbf{U}^{\mathrm{Me_6}}$ is −28.3 kcal/mol, while for $\mathbf{Np}^{\mathrm{Me_6}}$ the computed electron affinity is −21.9 kcal/mol. This is consistent with our prior analysis showing that the Np electronic structure is similar albeit less stable than the U-complex. On the other hand, the electron affinity for $\mathbf{Pu}^{\mathrm{Me_6}}$ is −16.7 kcal/mol. Therefore, the reduction becomes less favorable as one moves from $\mathbf{U}^{\mathrm{Me_6}}$ to $\mathbf{Pu}^{\mathrm{Me_6}}$, with theory suggesting that the Pu complexes are likely not stable and other ligand platforms should be considered.

5. Oxidation State Assignment

Table S15: LoProp charges on each of the B-atoms, computed from CASSCF-(9*e*,18*o*) calculations, for the quartet state of $\mathbf{U^{Me_6}}$, and with the (10*e*,18*o*) active space for the quintet and triplet state of $[\mathbf{U^{Me_6}}]^-$.

Complex	Spin	B1	B2	B3
U^{Me_6}	3/2	-0.5516	-0.5495	-0.5538
$[U^{Me_6}]^-$	2	-0.5815	-0.5847	-0.5866
$[U^{Me_6}]^-$	1	-0.5898	-0.5910	-0.5754

Table S16: LoProp charges on each of the B-atoms, computed from CASSCF-(10*e*,18*o*) calculations, for the quintet state of $\mathbf{Np^{Me_6}}$, and with the (11*e*,18*o*) active space for the sextet and quartet state of $[\mathbf{Np^{Me_6}}]^-$.

Complex	Spin	B1	B2	B3
Np^{Me_6}	2	-0.5499	-0.5500	-0.5500
$[Np^{Me_6}]^-$	5/2	-0.5811	-0.5685	-0.5852
$[Np^{Me_6}]^-$	3/2	-0.5685	-0.5781	-0.5801

Table S17: LoProp charges on each of the B-atoms, computed from CASSCF-(11*e*,18*o*) calculations, for the sextet state of $\mathbf{Pu^{Me_6}}$, and with the (12*e*,18*o*) active space for the septet and quintet state of $[\mathbf{Pu^{Me_6}}]^-$.

Complex	Spin	B1	B2	B3
Pu^{Me_6}	5/2	-0.5579	-0.5596	-0.5591
$[Pu^{Me_6}]^-$	3	-0.5899	-0.5943	-0.5945
$[Pu^{Me_6}]^-$	2	-0.5860	-0.5875	-0.5745

In the high-spin state of and $\mathbf{Pu^{Me_6^-}}$, a ligand-dominated δ-type orbital containing one electron is observed, suggesting that the additional electron goes to the arene, rather than the metal ion. However, in the lower spin states of both reduced congeners, where $5f-\pi$ mixing is more delocalized, determining the oxidation state of the metal ion is not straightforward. To evaluate the oxidation states, we analyzed the LoProp charges and Mulliken spin populations on the actinide ions and arene carbon atoms based on the CASSCF wavefunction (Tables S18 to S21).

For the Np complexes, LoProp charge analysis shows that in the neutral $\mathbf{Np^{Me_6}}$ complex, the metal ion has a partial positive charge of 2.44, and the arene has a partial charge of −0.17. Likewise,

Table S18: LoProp charge analysis from CASSCF-(10e,18o) for the quintet state of $\mathbf{Np^{Me_6}}$, and with (11e,18o) active space for the sextet and quartet state of $[\mathbf{Np^{Me_6}}]^-$.

Complex	Spin	Np	C1	C2	C3	C4	C5	C6	$\sum_{Arene}$
Np^{Me_6}	2	2.4418	-0.0179	-0.0432	-0.0180	-0.0389	-0.0174	-0.0390	-0.1744
$[Np^{Me_6}]^-$	5/2	2.4057	-0.2085	-0.0937	-0.0678	-0.2323	-0.0514	-0.0615	-0.7152
$[Np^{Me_6}]^-$	3/2	2.4270	-0.0584	-0.0743	-0.2399	-0.0760	-0.0572	-0.2413	-0.7471

Table S19: Mulliken spin population analysis from CASSCF-(10e,18o) for the quintet state of $\mathbf{Np^{Me_6}}$, and with (11e,18o) active space for the sextet and quartet state of $[\mathbf{Np^{Me_6}}]^-$.

Complex	Spin	Np	C1	C2	C3	C4	C5	C6	$\sum_{Arene}$
Np^{Me_6}	2	3.9732	-0.0023	0.0041	-0.0023	0.0044	-0.0021	0.0044	0.0062
$[Np^{Me_6}]^-$	5/2	4.1085	0.3170	0.0387	0.0410	0.2951	0.0853	0.0430	0.8201
$[Np^{Me_6}]^-$	3/2	3.4979	-0.0286	-0.0259	-0.1866	-0.0260	-0.0266	-0.1906	-0.4843

Table S20: LoProp charge analysis from CASSCF-(11e,18o) for the sextet state of $\mathbf{Pu^{Me_6}}$, and with (12e,18o) active space for the septet and quintet state of $[\mathbf{Pu^{Me_6}}]^-$.

Complex	Spin	Pu	C1	C2	C3	C4	C5	C6	$\sum_{Arene}$
Pu^{Me_6}	5/2	2.4642	-0.0162	-0.0355	-0.0161	-0.0373	-0.0208	-0.0434	-0.1693
$[Pu^{Me_6}]^-$	3	2.4008	-0.0526	-0.0892	-0.1776	-0.0673	-0.0765	-0.2234	-0.6866
$[Pu^{Me_6}]^-$	2	2.4317	-0.0554	-0.2359	-0.0559	-0.0725	-0.2438	-0.0753	-0.7388

Table S21: Mulliken spin population analysis from CASSCF-(11e,18o) for the sextet state of $\mathbf{Pu^{Me_6}}$, and with (12e,18o) active space for the septet and quintet state of $[\mathbf{Pu^{Me_6}}]^-$.

Complex	Spin	Pu	C1	C2	C3	C4	C5	C6	$\sum_{Arene}$
Pu^{Me_6}	5/2	4.9711	-0.0002	0.0012	-0.0015	0.0004	0.0021	0.0001	0.0021
$[Pu^{Me_6}]^-$	3	5.1289	0.0604	0.0471	0.3298	0.0076	0.1019	0.2516	0.7984
$[Pu^{Me_6}]^-$	2	4.6040	-0.0327	-0.2294	-0.0349	-0.0370	-0.2204	-0.0377	-0.5921

$\mathbf{U}^{Me_6}$ shows a partial positive charge of 2.45 on the metal ion and −0.19 on the ligand.[7] Upon reduction to $\mathbf{Np}^{Me_6}{}^-$, there is little change in the partial positive charge on the metal ion, but the arene gains a significant amount of negative charge (−0.74 for S=3/2) (Table S18). This is supported by Mulliken spin-population analysis (Table S19): In the neutral Np complex, the metal ion has a spin population of 3.97 (theoretical value 4), with no spin population on the arene. Upon reduction, in the S=3/2 state of $\mathbf{Np}^{Me_6}{}^-$, spin pairing leads to significant spin delocalization resulting in the spin on the metal ion (spin population 3.5) being antiferromagnetically coupled to the arene radical (spin population −0.48).

In the $\mathbf{Pu}^{Me_6}{}^-$ complexes. Although the arene remains planar in the S=3 state of $\mathbf{Pu}^{Me_6}{}^-$, both LoProp charge and Mulliken spin population analyses suggest that the additional electron accumulates on the arene instead of the metal ion (Tables S20 and S21). We note in passing that for spin states of $\mathbf{Np}^{Me_6}{}^-$ where the arene adopts an "open-book" conformation in S=3/2 state, the out-of-plane carbon atoms accumulate more negative charge and spin population compared to the planar carbon atoms. This same phenomenon is observed in the S=2 state of $\mathbf{Pu}^{Me_6}{}^-$ complex. Although the arene remains planar in the S=3 state of $\mathbf{Pu}^{Me_6}{}^-$, an inhomogeneous distribution of charge and spin is observed within the arene.

Similar to $\mathbf{U}^{Me_6}{}^-$, both the $\mathbf{Np}^{Me_6}{}^-$ and $\mathbf{Pu}^{Me_6}{}^-$ complexes should be described as An(III) ions ferromagnetically or antiferromagnetically coupled with the arene radical, depending on the spin state. However, in the intermediate-spin states, the An–arene interaction is stronger in $\mathbf{U}^{Me_6}{}^-$, resulting in a greater charge separation between the metal ion and the arene radical compared to $\mathbf{Np}^{Me_6}{}^-$ and $\mathbf{Pu}^{Me_6}{}^-$. In $\mathbf{U}^{Me_6}{}^-$, the metal ion carries a positive charge of +2.58, while the arene radical has a charge of −0.89.[7] This charge separation is decreased in $\mathbf{Np}^{Me_6}{}^-$ and $\mathbf{Pu}^{Me_6}{}^-$, where the metal ion holds a charge of +2.43 and the arene radical a charge of −0.74.

References

(1) Klamt, A.; Schüürmann, G. COSMO: a new approach to dielectric screening in solvents with explicit expressions for the screening energy and its gradient. *J. Chem. Soc., Perkin Trans. 2* **1993**, 799–805.

(2) Bader, R. F. W. A Bond Path: A Universal Indicator of Bonded Interactions. *J. Phys. Chem. A* **1998**, *102*, 7314–7323.

(3) Bianchi, R.; Gervasio, G.; Marabello, D. Experimental Electron Density Analysis of $Mn_2(CO)_{10}$: Metal-Metal and Metal-Ligand Bond Characterization. *Inorg. Chem.* **2000**, *39*, 2360–2366.

(4) Lu, T.; Chen, F. Multiwfn: A Multifunctional Wavefunction Analyzer. *J. Comp. Chem.* **2012**, *33*, 580–592.

(5) Malmqvist, P. Å.; Roos, B. O.; Schimmelpfennig, B. The Restricted Active Space (RAS) State Interaction Approach with Spin–Orbit Coupling. *Chem. Phys. Lett.* **2002**, *357*, 230–240.

(6) Li Manni, G. et al. The OpenMolcas Web: A Community-Driven Approach to Advancing Computational Chemistry. *J. Chem. Theory Comput.* **2023**, *19*, 6933–6991.

(7) Chowdhury, S. R.; Goodwin, C. A. P.; Vlaisavljevich, B. What is the Nature of the Uranium(III)–Arene Bond? *Chem. Sci.* **2024**, *15*, 1810–1819.
